# Identification and Functional Analysis of Potato Receptor Kinase RDA2 Proteins

**DOI:** 10.3390/plants15060906

**Published:** 2026-03-14

**Authors:** Xuefeng Fan, Yue Han, Xiaoyu Sun, Tongjun Sun

**Affiliations:** 1 Shenzhen Branch, Guangdong Laboratory for Lingnan Modern Agriculture, Genome Analysis Laboratory of the Ministry of Agriculture and Rural Affairs, Agricultural Genomics Institute at Shenzhen, Chinese Academy of Agricultural Sciences, Shenzhen 518000, China; fanxuefeng@caas.cn (X.F.); 13383915568@163.com (Y.H.); sunxy2026@163.com (X.S.); 2 College of Agronomy, Shanxi Agricultural University, Jinzhong 030801, China

**Keywords:** StRDA2, *Phytophthora*, disease resistance, plant immunity

## Abstract

Plants utilize cell surface pattern recognition receptors to recognize pathogen-associated molecular patterns (PAMPs) and activate pattern-triggered immunity (PTI) responses. Late blight, caused by the oomycete plant pathogen *Phytophthora infestans*, poses a major threat to global potato production. The oomycete PAMP, *P. infestans* cell wall ceramide D, triggers reactive oxygen species (ROS) production in potato and *Arabidopsis*. It is specifically recognized by the lectin receptor-like kinase RESISTANT TO DFPM-INHIBITION OF ABSCISIC ACID SIGNALING 2 (RDA2) in *Arabidopsis*. Treatment with *P. infestans* ceramide D enhances potato resistance against *P. infestans*. However, the function of RDA2 homologs in potato remains uncharacterized. Herein, potato *RDA2* genes were identified through sequence alignment analysis. Their expression levels were subsequently measured in a potato inbred line infected with *P. infestans*. Notably, transient expression of *StRDA2A*, but not its kinase-dead mutant *StRDA2A^K543M^*, caused cell death and enhanced disease resistance in *Nicotiana benthamiana*. Additionally, two RXLR-type effector proteins significantly inhibited StRDA2A-induced cell death. The findings of this study suggest that potato receptor kinase RDA2 proteins confer disease resistance, which is attenuated by RXLR effectors secreted by *P. infestans*.

## 1. Introduction

Plants rely on a multilayered immune system to defend against invading pathogens. Cell surface pattern recognition receptors (PRRs) perceive conserved pathogen-associated molecular patterns (PAMPs) and activate pattern-triggered immunity (PTI). In contrast, intracellular nucleotide-binding leucine-rich repeat (NLR) proteins recognize pathogen effectors, thereby inducing effector-triggered immunity (ETI) [1]. Activation of PRRs initiates downstream signaling events, including the production of reactive oxygen species (ROS), calcium influx, and mitogen-activated protein kinase (MAPK) cascades, ultimately leading to transcriptional reprogramming and defense responses [2].

Lectin receptor-like kinases (LecRLKs) constitute a large and diverse subclass of PRRs characterized by extracellular lectin domains capable of binding carbohydrate- and lipid-derived molecules [3]. Notably, LecRLKs play critical roles in plant immunity by sensing pathogen-associated glycans and lipids. For example, the *Arabidopsis* G-type LecRLK LIPOOLIGOSACCHARIDE-SPECIFIC REDUCED ELICITATION (LORE) recognizes medium-chain 3-hydroxy fatty acids derived from bacterial lipopolysaccharides [4]. In contrast, other LecRLKs participate in immune signaling against insects and microbial pathogens [5]. These studies underscore the importance of LecRLKs, expanding recognition capacity in plant immunity beyond classical proteinaceous elicitors.

Recently, the *Arabidopsis* lectin receptor kinase RDA2 (RESISTANT TO DFPM-INHIBITION OF ABSCISIC ACID SIGNALING 2) was identified as a PRR recognizing the oomycete-derived 9-methyl-branched sphingoid base (9Me-Spt), which originates from *Phytophthora infestans* ceramide D (Pi-Cer D) [6]. RDA2 activation initiates immune responses. Notably, loss-of-function mutations in either RDA2 or neutral ceramidase 2 (NCER2) significantly compromise *Arabidopsis* resistance to oomycete pathogens [6]. Besides its role in immunity, RDA2 mediates crosstalk between pathogen defense and abscisic acid (ABA) signaling, antagonizing ABA-mediated stress responses that would otherwise suppress immune activation [7]. However, whether RDA2-mediated immune mechanisms are conserved in crop species remains unexplored.

Late blight, caused by *P. infestans*, continues to pose a severe threat to global potato production. Numerous intracellular NLR resistance genes have been identified and deployed in breeding programs [8]. However, comparatively little is known about cell surface PRRs involved in potato immunity, particularly LecRLKs. This gap is important because oomycetes produce abundant lipid- and glycan-derived molecules, which are potential ligands for LecRLKs. However, it remains to be determined whether the potato genome encodes functional RDA2 homologs.

Successful *P. infestans* infection relies on the secretion of hundreds of RXLR effectors that suppress host immune responses [9]. These effectors target diverse components of PTI and ETI signaling, including MAPK cascades and regulators of programmed cell death. Notably, several RXLR effectors interfere with receptor-mediated signaling and plasma membrane-associated immune processes, suggesting that PRR-triggered pathways represent prominent effector targets [10]. However, it remains unknown whether RXLR effectors suppress RDA2-mediated immune signaling.

In this study, four RDA2 homologs were identified in the diploid potato inbred line A157. Their expression patterns and immune functions were subsequently investigated. Notably, StRDA2A and StRDA2D induced hypersensitive cell death and enhanced resistance to *Phytophthora* species when transiently expressed in *Nicotiana benthamiana*. Moreover, StRDA2A activated MAPK signaling in a kinase-dependent manner. Its kinase activity was essential for cell death induction and disease resistance when transiently expressed in *N. benthamiana*. Several RXLR effectors that suppressed StRDA2A-mediated immune responses were also identified. These results collectively reveal that potato RDA2 proteins are functional immune receptors and provide a molecular framework for exploiting LecRLK-mediated resistance in potato breeding programs.

## 2. Results

### 2.1. Identification and Expression Characteristics of StRDA2 Genes in Potato

**Figure 1:**
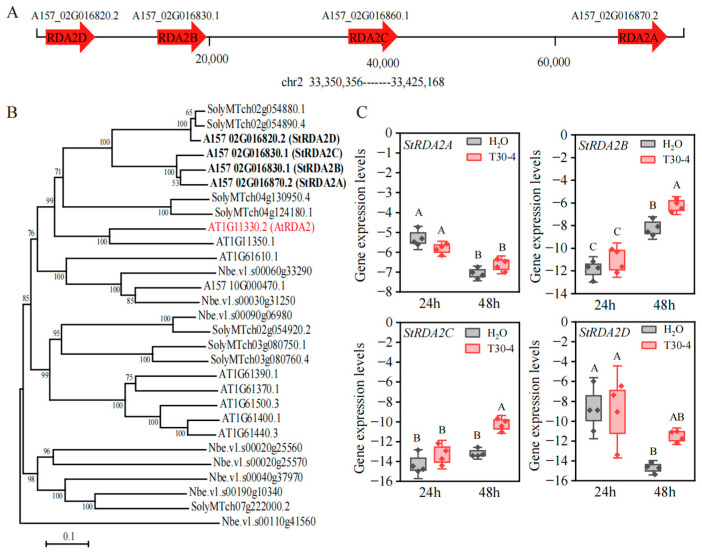
Genomic distribution and expression patterns of RDA2 genes in potatoes. (**A**) Genomic distribution of *StRDA2A*, *StRDA2B*, *StRDA2C*, and *StRDA2D* on chromosome 2 of *Solanum tuberosum* (cv. A157). Physical distances between adjacent genes (bp) and the chromosome region (chr2: 33,350,356–33,452,168) are indicated. (**B**) Phylogenetic analysis of *S. tuberosum* RDA2 (StRDA2) proteins and their homologs from *Arabidopsis thaliana*, *Nicotiana benthamiana*, and *S. lycopersicum*. The phylogenetic tree was constructed using the maximum-likelihood method based on amino acid sequence alignments with 1000 bootstrap replicates. Bootstrap values >50% are shown. (**C**) The relative expression levels of *SRDA2A*, *StRDA2B*, *StRDA2C*, and *StRDA2D* in *S. tuberosum* leaves treated with mock solution (H_2_O) and those inoculated with *P. infestans* strain T30-4 at 24 and 48 h post-treatment. Transcript levels were determined by qRT-PCR and normalized based on levels of *StEF1α*. Data represent the mean ± SD of three independent biological replicates. Different letters denote statistically significant differences (Tukey’s HSD test, *p* < 0.05).

In a previous study, three potato proteins (XP_015164178.1, XP_015164180.1, and XP_015164179.1) in cv. DM exhibited high sequence similarity to *A. thaliana* RDA2 (AtRDA2) [6]. Herein, these proteins were used as queries for BLASTp 2.12.0+ searches against the A157 genome database (http://solomics.agis.org.cn/potato), accessed on 8 April 2024, to systematically identify RDA2 homologs in the diploid potato inbred line A157. The search yielded four RDA2-related genes: *StRDA2A* (A157_02G016870.2), *StRDA2B* (A157_02G016860.1), *StRDA2C* (A157_02G016830.1), and *StRDA2D* (A157_02G016820.2). Notably, these genes were clustered within an approximately 96-kb region on chromosome 2 (33.356–33.452 Mb; Figure 1A), suggesting that they might have arisen through local gene duplication events.

A phylogenetic tree was constructed using MEGA11 based on sequences of homologs from *A. thaliana*, *N. benthamiana*, *S. lycopersicum* (cv. MicroTOM), and *S. tuberosum* (inbred line A157) to examine the evolutionary relationships among the RDA2 proteins. Notably, RDA2 proteins from the families Solanaceae (potato and tomato) and Brassicaceae (*Arabidopsis*) formed a well-supported monophyletic clade, suggesting strong evolutionary conservation across these lineages (Figure 1B). Gene structure analysis revealed that the four *StRDA2* genes each contained seven exons and six introns (Supplementary Figure S1A). Coding sequence (CDS) alignment showed that *StRDA2B* and *StRDA2C* share more than 90% sequence similarity with *StRDA2A*, whereas *StRDA2D* exhibits approximately 76% identity with the other three paralogs (Supplementary Figure S1B). At the protein level, potato StRDA2 proteins share approximately 50% amino acid identity with *Arabidopsis* RDA2. Among the potato paralogs, StRDA2A, StRDA2B, and StRDA2C were determined to be highly conserved, with around 85% amino acid identity, whereas StRDA2D was more divergent, showing only ~64% identity relative to the other three members (Supplementary Figure S1C). In summary, the StRDA2 gene family displays conserved exon–intron architecture but clear sequence divergence among paralogs, particularly distinguishing StRDA2D from the other three highly similar members.

The expression levels of *StRDA2* genes were examined in potato leaves inoculated with *P. infestans* in order to examine whether they were transcriptionally regulated during pathogen infection. Notably, the transcript levels of *StRDA2A* and *StRDA2D* were not significantly induced at 24 and 48 h post-inoculation compared to the mock-treated samples. In contrast, *StRDA2B* and *StRDA2C* exhibited a modest but statistically significant increase in transcript levels at 48 h post-inoculation compared to the mock treatment. (Figure 1C). These results suggested that *StRDA2* genes were not strongly induced by *P. infestans* infection under the tested conditions.

### 2.2. StRDA2 Proteins Trigger Cell Death Independently of Salicylic Acid Biosynthesis

**Figure 2:**
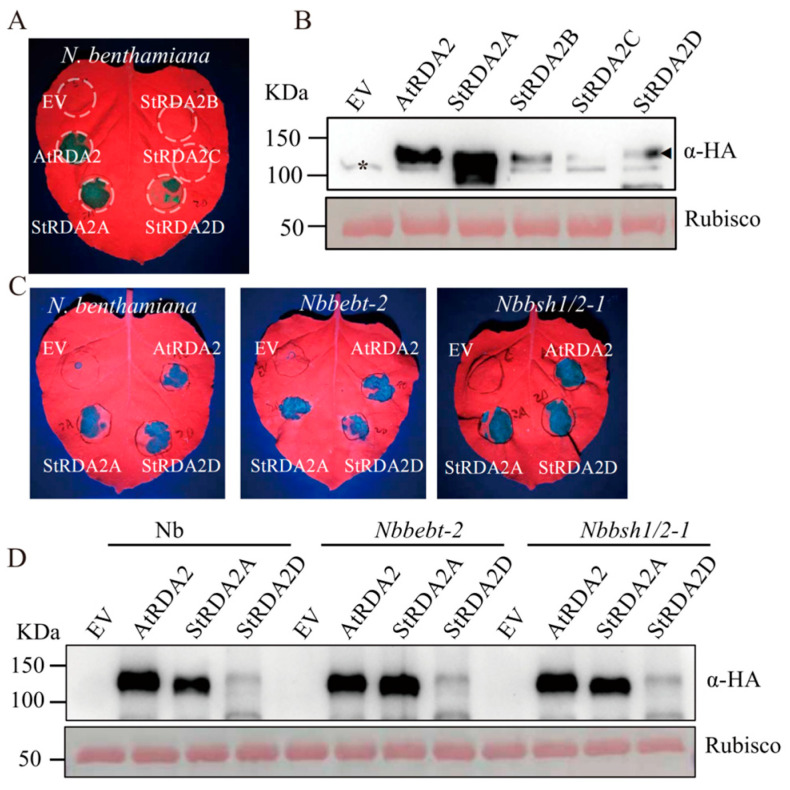
Transient expression of *AtRDA2* and *StRDA2* homologs induced cell death in *Nicotiana benthamiana* independent of salicylic acid. (**A**) Cell death phenotypes in *N. benthamiana* leaves at 48 h post-treatment (hpt) following transient expression of *AtRDA2*, *StRDA2A*, *StRDA2B*, *StRDA2C*, and *StRDA2D*. The empty vector (EV) was used as the negative control. (**B**) Accumulation of AtRDA2 and StRDA2 proteins in *N. benthamiana* leaves at 24 h post-transient expression. Proteins were detected by immunoblotting using an anti-HA antibody. Rubisco, visualized by Ponceau S staining, served as the loading control. Arrowheads indicate the target RDA protein bands, while the asterisk (*) marks a non-specific band. (**C**) Cell death phenotypes observed in *N. benthamiana* and salicylic acid-deficient mutant lines (*Nbbebt-2* and *Nbbsh1/2-1*) at 48 hpt. (**D**) Protein accumulation of RDA2 family members in *Nbbebt-2* and *Nbbsh1/2-1* plants at 24 hpt, corresponding to (**C**).

*AtRDA2* and the four *StRDA2* homologs were transiently expressed in *N. benthamiana* leaves via *Agrobacterium*-mediated infiltration to investigate whether StRDA2 proteins could induce immune-associated cell death. Visible cell death was observed in leaves expressing *AtRDA2*, *StRDA2A*, and *StRDA2D* at 48 h post-infiltration (hpi). In contrast, no necrotic symptoms were observed in leaves expressing *StRDA2B*, *StRDA2C,* and the empty vector (EV) control (Figure 2A).

Immunoblot analysis was performed at 24 hpt to exclude the possibility that the observed phenotypes were caused by differences in protein accumulation. Notably, all RDA2 proteins were detected at appreciable levels, confirming successful expression of the constructs (Figure 2B). AtRDA2 and StRDA2A accumulated at higher levels than the other homologs. StRD2A and StRD2D induced comparable cell death phenotypes, indicating that protein abundance did not strictly determine the strength of immune activation among RDA2 paralogs.

To determine whether salicylic acid (SA) signaling is required for RDA2-triggered cell death, *AtRDA2*, *StRDA2A*, and *StRDA2D* were transiently expressed in *N. benthamian*a and in SA-deficient mutant lines (*Nbbebt-2* and *Nbbsh1/2-1*) [11]. Robust cell death symptoms were observed in both wild-type plants and SA-deficient backgrounds (Figure 2C). SA-deficient *NahG*-transgenic *N. benthamian*a also exhibited cell death following the expression of *AtRDA2*, *StRDA2A*, and *StRDA2D* (Supplementary Figure S2). Immunoblot analysis confirmed that the accumulation level of RDA2 proteins in wild-type and SA-deficient plants was similar (Figure 2D). These results collectively demonstrated that RDA2-mediated cell death occurred independently of salicylic acid signaling.

### 2.3. StRDA2A and StRDA2D Enhance Resistance to Phytophthora Species

**Figure 3:**
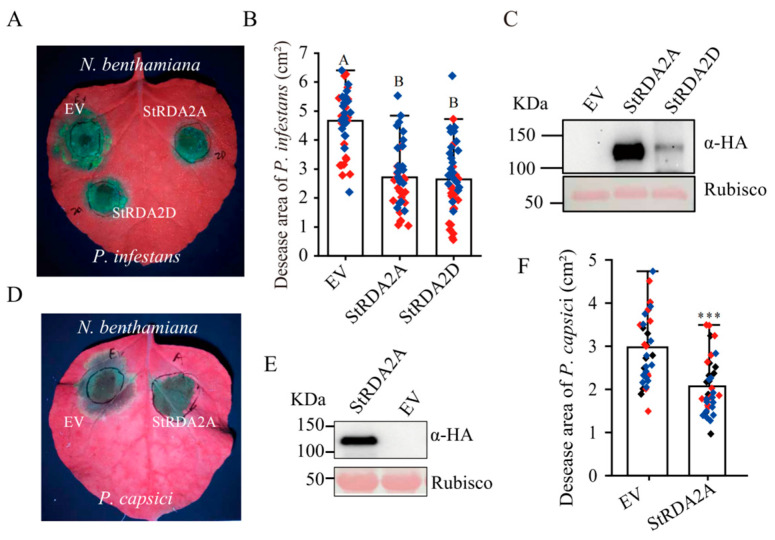
StRDA2A and StRDA2D enhanced *Nicotiana benthamiana* resistance to *Phytophthora* species. (**A**) Disease symptoms of *N. benthamiana* leaves at 5 days post-inoculation (dpi) with *P. infestans*. The empty vector (EV) served as the negative control. (**B**) Quantification of lesion areas caused by *P. infestans*. Lesion sizes were measured using ImageJ 1.51j8 software. Data were derived from two independent experiments. Statistically significant differences are indicated by labeling with different letters (Tukey’s HSD test, *p* < 0.05, *n* = 42). Data points with the same color originate from the same biological replicate. (**C**) Protein accumulation of StRDA2A and StRDA2D in *N. benthamiana* leaves at 24 h post-agroinfiltration, corresponding to the *P. infestans* inoculation assay. (**D**) Disease phenotypes of *N. benthamiana* leaves at 3 dpi following *P. capsici* inoculation. (**E**) Protein accumulation of StRDA2A in *N. benthamiana* leaves at 24 h post-agroinfiltration, corresponding to the *P. capsici* infection assay. (**F**) Quantification of lesion areas caused by *P. capsici.* Lesion sizes were measured using ImageJ software. Data were derived from three independent experiments (Student’s *t*-test, *** *p* < 0.001, *n* = 36).

*N. benthamiana* leaves transiently expressing *StRDA2A* and *StRDA2D* were challenged with *Phytophthora* pathogens to evaluate whether StRDA2 proteins contribute to disease resistance. Leaves transiently expressing *StRDA2A* and *StRDA2D* were inoculated with *P. infestans*, followed by an evaluation of the disease symptoms at 5 days post-inoculation (dpi). Notably, leaves expressing either *StRDA2A* or *StRDA2D* exhibited a significant reduction in lesion sizes compared to those containing the empty vector (EV) (Figure 3A). Quantification of lesion areas confirmed that *P. infestans*-induced disease symptoms were significantly reduced in leaves expressing *StRDA2A* or *StRDA2D* (Figure 3B). Immunoblot analysis performed at 24 h post-agroinfiltration revealed that both proteins accumulated prior to pathogen inoculation (Figure 3C).

Leaves expressing StRDA2A were inoculated with *P. capsici* strain LT263 to examine whether the resistance conferred by StRDA2A extends to other *Phytophthora* species. Notably, disease symptoms were visibly reduced compared with the EV control at 3 dpi (Figure 3D). Stable accumulation of StRDA2A protein was observed prior to pathogen inoculation (Figure 3E). Moreover, quantitative measurements revealed a significant reduction in lesion size (Figure 3F). These results indicated that StRDA2A and StRDA2D positively regulated resistance to multiple *Phytophthora* species.

### 2.4. Kinase Activity of StRDA2A Is Required for Cell Death Induction and Disease Resistance

**Figure 4:**
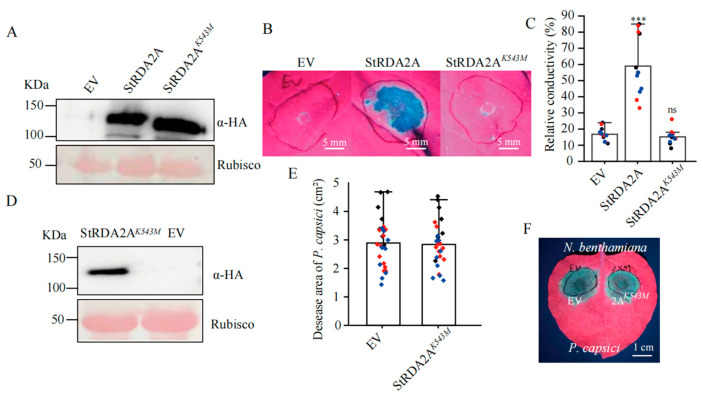
Kinase activity of StRDA2A is required for cell death induction and disease resistance. (**A**) Protein accumulation of StRDA2A and its kinase-inactive mutant StRDA2A*^K543M^* in *Nicotiana benthamiana* leaves at 24 h post-treatment. (**B**) Cell death phenotypes observed at 48 h post-infiltration. Leaves expressing *StRDA2A* induced strong cell death, whereas those expressing *StRDA2A^K543M^* or EV did not. Images were captured under UV light. Scale bar = 5 mm. (**C**) Quantification of cell death by electrolyte leakage assay. Data were derived from three independent biological replicates (Student’s *t*-test, *** *p* < 0.001; ns, not significant; *n* = 12). Data points with the same color originate from the same biological replicate. (**D**) Immunoblot analysis results confirming the accumulation of StRDA2AK543M in *N*. *benthamiana* leaves at 24 h post-infiltration. Rubisco (Ponceau S staining) served as the loading control. (**E**) Quantification of lesion area following *P. capsici* inoculation. Leaves were inoculated with either EV or StRDA2AK543 M at 16 h post-agroinfiltration. Lesion sizes were measured using ImageJ 1.51j8 software. No significant differences were detected (Student’s *t*-test, *p* = 0.562, *n* = 30). Data points shown in the same color were derived from the same biological experiment. (**F**) Disease phenotypes visualized under UV light at 48 h post-*P. capsici* inoculation. EV and StRDA2A*^K543M^* exhibited comparable disease severity. Scale bar = 1 cm.

Sequence alignment analysis revealed that Lys^543^ is a conserved residue within the ATP-binding site of the StRDA2A kinase domain, which is essential for receptor kinase activity (Supplemental Figure S3). A putative kinase-dead mutant was thus generated by substituting Lys^543^ with methionine (StRDA2A*^K543M^*) to assess the requirement of kinase activity. Notably, wild-type StRDA2A and the mutant protein accumulated to comparable levels in *N. benthamiana* leaves at 24 h post-transient expression (Figure 4A), indicating that the K543M mutation did not affect protein stability.

Leaves expressing wild-type StRDA2A exhibited cell death, whereas those expressing StRDA2A*^K543M^* or EV showed no visible necrosis at 48 h post infiltration (Figure 4B). Quantitative electrolyte leakage assays further confirmed significantly reduced ion leakage in tissues expressing the kinase-dead mutant compared to those expressing wild-type StRDA2A (Figure 4C). These results indicated that the kinase activity of StRDA2A is essential for its ability to trigger cell death.

*N. benthamiana* leaves expressing StRDA2A*^K543M^* or EV were inoculated with *P. capsici* at 16 h post-agroinfiltration to examine whether the kinase activity of StRDA2A was essential in inducing disease resistance. Immunoblot analysis confirmed stable accumulation of StRDA2A*^K543M^* prior to pathogen challenge (Figure 4D). Comparable disease symptoms and lesion sizes were observed between StRDA2A*^K543M^*-expressing leaves and EV controls (Figure 4E,F). These results indicated that kinase activity was essential for StRDA2A-mediated cell death induction and disease resistance.

### 2.5. StRDA2A Activates MAPK Signaling Cascade in a Kinase-Dependent Manner

**Figure 5:**
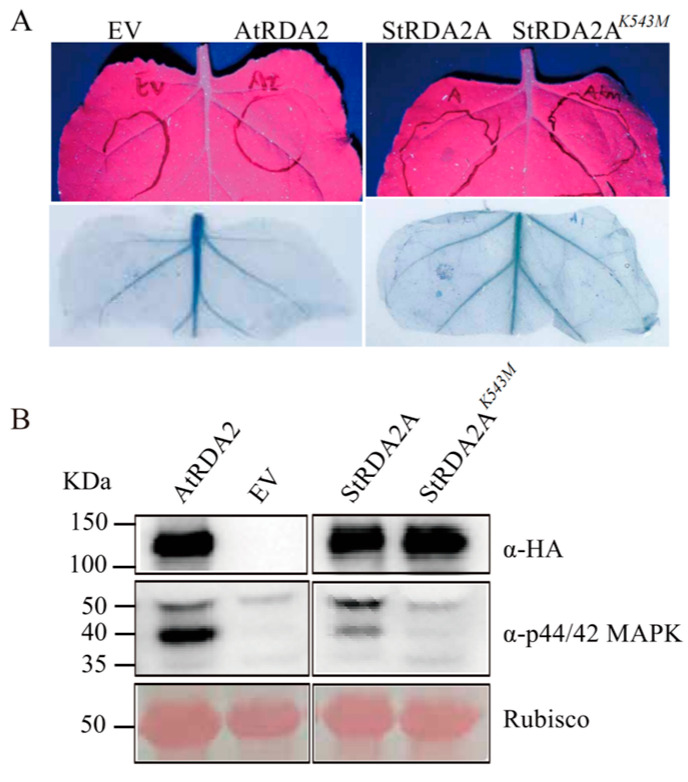
StRDA2A activates MAPK signaling in a kinase activity-dependent manner. (**A**) Cell death phenotypes in *Nicotiana benthamiana* leaves at 24 h post-treatment following the expression of *AtRDA2*, *StRDA2D*, *StRDA2A*, and *StRDA2Aᴷ^543^ᴹ*. The empty vector (EV) served as the negative control. The upper panel shows images under UV light, while the lower panels show trypan blue staining results. (**B**) Immunoblot analysis of MAPK activation. Phosphorylated MPK3/MPK6 was detected using an anti-p44/42 MAPK antibody. Accumulation of RDA2 proteins was confirmed using an anti-HA antibody. Rubisco served as the loading control.

Mitogen-activated protein kinase (MAPK) cascades are central components of plant immune signaling. *AtRDA2*, *StRDA2A*, *StRDA2A^K543M^*, and EV were transiently expressed in *N. benthamiana* to determine whether StRDA2A activates MAPK signaling. Notably, no visible cell death was detected at 24 h post-infiltration, allowing MAPK activation to be examined independently of hypersensitive responses (Figure 5A). Immunoblot analysis revealed strong phosphorylation of MPK3 and MPK6 in leaves expressing *AtRDA2* or *StRDA2A*. In contrast, no detectable MAPK activation was observed in tissues expressing *StRDA2A^K543M^* or the empty vector control (Figure 5B). Protein accumulation of all constructs was confirmed at the same time point. These results indicated that StRDA2A activates MAPK signaling in a kinase-dependent manner.

### 2.6. Phytophthora RXLR Effectors Suppress StRDA2A-Mediated Cell Death

**Figure 6:**
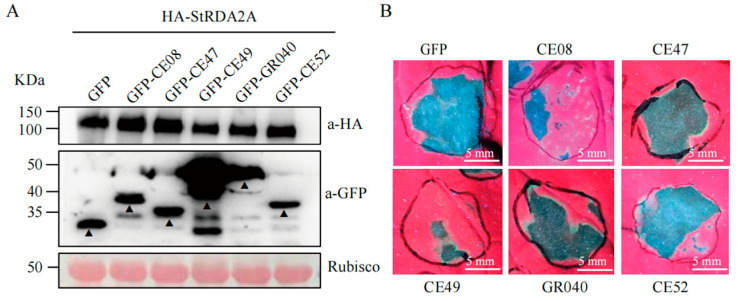
*Phytophthora* RXLR effectors suppress StRDA2A-induced cell death. (**A**) Immunoblot analysis of protein accumulation at 24 h post-agroinfiltration. StRDA2A was detected using an anti-HA antibody, while GFP-tagged RXLR effectors were detected using an anti-GFP antibody. Rubisco, visualized by Ponceau S staining, served as the loading control. Arrowheads indicate the RXLR-GFP fusion protein bands. (**B**) Suppression of StRDA2A-induced cell death by RXLR effectors in *Nicotiana benthamiana* leaves. Images were captured under UV light at 3 days post-agroinfiltration (dpi). GFP served as the negative control. Scale bar = 5 mm.

Cell death inducers, such as BAX, INF1, NIP, Avh238, and Avh241, activate plant PTI and ETI defenses and are commonly used to examine the virulence functions of effectors [12]. In the present study, five RXLR effectors, including CE49, CE08, GR040, CE52, and CE47, which can suppress cell necrosis induced by a range of cell death inducers, including INF1, BAX, and NIP, were analyzed (details in Table 1). These RXLR effectors were co-expressed with StRDA2A in *N. benthamiana* to determine whether *Phytophthora* RXLR effectors interfere with StRDA2A-mediated immunity. Immunoblot analysis confirmed comparable accumulation of StRDA2A in all treatments (Figure 6A). Phenotypic observation at two days post-infiltration showed that all five RXLR effectors suppressed StRDA2A-induced cell death to varying degrees compared with the GFP control (Figure 6B). CE49 exhibited the strongest inhibitory activity, with an inhibition rate of 69.7%, followed by CE08 at 45.7%. GR040, CE52, and CE47 displayed weaker suppression, with inhibition rates of 30.4%, 23.3%, and 10.9%, respectively. (Detailed information on these effectors is provided in Table 1). These results demonstrated that *Phytophthora* RXLR effectors could attenuate StRDA2A-triggered cell death, suggesting that StRDA2A-mediated immune signaling is a common target of pathogen effectors during infection.

## 3. Discussion

In this study, the *RDA2* gene family was systematically characterized in potato. Notably, specific members were determined to function as immune regulators against *Phytophthora* species. StRDA2A and StRDA2D exhibited clear immune activity when transiently expressed in *N. benthamiana*, as evidenced by the induction of hypersensitive cell death and enhanced resistance to oomycete pathogens. These findings corroborate previous research on RDA2 in *A. thaliana*, which identified RDA2 as a lectin receptor-like kinase involved in sphingolipid-mediated immune signaling [6], indicating that RDA2-mediated immune signaling is conserved beyond *Arabidopsis*.

Phylogenetic analysis revealed that RDA2 homologs from *Solanaceae* and *Brassicaceae* formed a conserved clade. This finding suggested that this receptor family had an ancient evolutionary origin. Gene duplication and retention within this clade may have contributed to functional diversification, a common feature observed among receptor-like kinases involved in pathogen perception [16]. The four potato *RDA2* genes exhibited distinct expression levels and functional behaviors despite this conservation. Notably, none of the *StRDA2* genes was strongly transcriptionally induced upon *P. infestans* infection. This feature is consistent with many PRRs, whose activities are mainly controlled through post-transcriptional mechanisms, such as phosphorylation [17], receptor complex formation, and protein turnover. The relatively low accumulation of StRDA2D protein further implies that individual paralogs may be subject to different post-translational regulation mechanisms, potentially reflecting functional diversification following gene duplication.

Notably, kinase activity was essential for StRDA2A-mediated immune responses. Mutation of the conserved ATP-binding lysine completely attenuated cell death induction and MAPK activation and enhanced disease resistance, indicating that StRDA2A functions as an active signaling kinase rather than a passive scaffold. Similar kinase-dependent immune signaling has been reported for multiple receptor-like kinases involved in pattern-triggered immunity, such as FLS2-associated signaling complexes [18] and CERK1-mediated pathways [19]. This requirement parallels that of many receptor-like kinases involved in PTI, in which trans-phosphorylation between coreceptors and the kinase domain of PRRs (or adapter kinases like SOBIR1) is recognized as the initial intracellular signaling event in response to PAMPs [20]. Accordingly, StRDA2A, but not its kinase-dead variant, activated MAPK signaling, placing MAPK activation downstream of StRDA2A function. These observations supported a model in which StRDA2A functions as an upstream regulator that initiates kinase-dependent signaling cascades, leading to defense activation. Although transient expression in *N. benthamiana* provides a powerful system to dissect immune signaling, it occurs in a heterologous context. Functional validation of StRDA2A in stable transgenic or genome-edited potato plants is thus essential to establish its contribution to resistance under natural infection conditions. Moreover, future studies should decipher whether StRDA2 proteins directly perceive oomycete-derived sphingolipids, as reported for *Arabidopsis* RDA2 [6].

Notably, two RXLR effectors that significantly suppressed StRDA2A-mediated cell death were identified. RXLR effectors target crucial nodes of host immune signaling, including MAPK cascades, transcriptional regulators, and cell death execution machinery [21]. The observation that multiple RXLR effectors attenuate StRDA2A-triggered responses suggests that this receptor-mediated pathway constitutes a relevant target during infection. Functional redundancy among RXLR effectors has been widely documented and is thought to enhance pathogen adaptability and robustness in suppressing host immunity [13]. However, the precise molecular targets of these RXLR effectors remain unknown.

The present study revealed that potato RDA2 proteins, particularly StRDA2A, function as immune receptors that activate MAPK signaling and confer resistance to *Phytophthora* species in a kinase-dependent manner. The ability of RXLR effectors to suppress RDA2-mediated immunity underscores the importance of this pathway as a critical battleground in the host–pathogen arms race. These findings expand our understanding of lectin receptor kinase-mediated defense mechanisms in crops. Moreover, they highlight the potential utility of PRR-based strategies for improving disease resistance against oomycete pathogens.

## 4. Materials and Methods

### 4.1. Plant Materials and Growth Conditions

*Nicotiana benthamiana* plants were grown in a growth chamber set at 25 °C with a 16 h/8 h light/dark cycle. *N. benthamiana* seeds were first germinated in a sterilized soil mixture consisting of peat moss, vermiculite, and perlite (3:1:1, *v*:*v*:*v*). Two-week-old seedlings were transplanted into individual pots and grown for an additional 2–3 weeks before use. Salicylic acid-deficient mutant lines (*Nbbebt-2* and *Nbbsh1/2-1*) were provided by Dr. Zhang [11]. The diploid potato (*S. tuberosum* L.) inbred line A157 (accession A6-26) [22] was maintained in the laboratory and cultivated under identical environmental conditions.

*Escherichia coli* DH5α cells were used for plasmid propagation, while *Agrobacterium tumefaciens* GV3101 was used for plant transient expression assays. *Phytophthora capsici* strain LT263 and *P. infestans* strain 1306 were maintained on V8 agar medium.

### 4.2. Plasmid Construction

The full-length coding sequences (CDSs) of *AtRDA2*, *StRDA2A*, *StRDA2B*, *StRDA2C*, and *StRDA2D* (listed in Supplementary Text S1) were amplified by PCR and cloned into the binary expression vector pCAMBIA1305-35S-3HA carrying a CaMV 35S promoter and C-terminal 3 × HA epitope tag. Vector linearization was performed using *Hin*dIII and *Kpn*I (New England Biolabs, Ipswich, MA, USA), followed by homologous recombination-based cloning. The conserved ATP-binding lysine residue (Lys^543^) was substituted with methionine using site-directed mutagenesis, producing StRDA2A*^K543M^*, a kinase-inactive mutant.

The coding sequences of RXLR effectors (CE08, CE47, CE49, GR040, and CE52) were cloned into the binary vector pMDC-35S-GFP to generate N-terminal GFP fusion proteins under the control of the CaMV 35S promoter. Vector backbones were linearized for recombination using *Spe*I and *Sac*I (New England Biolabs, Ipswich, MA, USA). All primers used were designed using SnapGene v6.0.2 and synthesized by Tsingke Biotechnology Co., Ltd. (Beijing, China). The primer sequences are listed in Supplementary Table S1.

### 4.3. Agrobacterium-Mediated Transient Expression in N. benthamiana

Recombinant *A. tumefaciens* GV3101 strains were cultured overnight in LB medium supplemented with kanamycin (50 μg/mL), rifampicin (25 μg/mL), and gentamycin (50 μg/mL) in an incubator shaker set at 28 °C and 200 rpm. The culture was then centrifuged at 5000 rpm for 5 min to harvest the bacterial cells, which were subsequently washed twice with infiltration buffer (10 mM MgCl_2_, 10 mM MES, pH = 5.6, 200 μM acetosyringone). The bacterial cells were resuspended in infiltration buffer and adjusted to an OD_600_ of 0.6. Cell suspensions were incubated at room temperature in the dark for 3 h to induce the expression of virulence genes. Equal volumes of *Agrobacterium* cultures harboring different constructs were mixed prior to co-infiltration.

Fully expanded leaves of 4–5-week-old *N. benthamiana* plants were infiltrated with the cell suspensions on the abaxial side using a needleless syringe. The plants were subsequently maintained under low light conditions for 24 h and then returned to standard growth conditions. Leaf samples were collected at 24 h post-infiltration (dpi) for protein detection and again at 48 dpi for phenotype observation (i.e., cell necrosis observation).

### 4.4. Protein Extraction and Immunoblot Analysis

Leaf tissue samples (approximately 50 mg) from infiltrated regions were harvested, frozen in liquid nitrogen, and ground into a fine powder. The ground samples were dissolved in 100 μL of 2 × SDS loading buffer (100 mM Tris-HCl, pH 6.8, 4% SDS, 20% glycerol, 0.2% bromophenol blue, 10% β-mercaptoethanol), boiled at 95 °C for 10 min, and then centrifuged at 12,000× *g* for 10 min to obtain the proteins (supernatant). Proteins were separated by 8% SDS-PAGE and subsequently transferred onto PVDF membranes (Millipore, Billerica, MA, USA) using a semi-dry blotting system (1.5A for 10 min). Membranes were blocked with 5% (*w*/*v*) non-fat milk in TBST buffer (20 mM Tris-HCl, pH 7.5, 150 mM NaCl, 0.1% Tween-20) for 45 min at room temperature, followed by overnight incubation at 4 °C with primary antibodies.

Anti-HA antibody (mouse monoclonal, 1:2000 dilution; ab1424, Abcam, Cambridge, UK) was used to detect HA-tagged RDA2 proteins, while anti-GFP antibody (rabbit monoclonal, 1:2000 dilution; ab290, Abcam) was used to detect GFP-tagged RXLR effectors. The PVDF membranes were washed and subsequently incubated with horseradish peroxidase (HRP)-conjugated secondary antibodies (anti-mouse and anti-rabbit IgG-HRP, 1:5000 dilution; LF101 or LF102, YamayBio, San Diego, CA, USA) for 1 h at room temperature. Protein signals were visualized using an enhanced chemiluminescence (ECL) detection system (E171-04, GenStar, Beijing, China) and imaged with a ChemiDoc MP imaging system (Bio-Rad, Hercules, CA, USA). Ponceau S staining of Rubisco (large subunit, approximately 55 kDa) was used as a loading control.

### 4.5. Electrolyte Leakage Assay

Cell death was quantified by measuring electrolyte leakage. Three leaf discs (1.5 cm diameter) were excised from the infiltrated areas 2 days post-infiltration (dpi) and floated in 3 mL of deionized water at room temperature for 3 h with gentle shaking. Initial conductivity (R1) was measured using a conductivity meter (DDS-307, LeiCi, Shanghai, China). The leaf discs were then boiled at 100 °C for 30 min to release the total electrolytes and cooled to room temperature, after which the final conductivity (R2) was measured. The relative electrolyte leakage (REL) was calculated using the following formula:

REL (%) = (R1/R2) × 100%.

### 4.6. Phytophthora Inoculation Assays

*Phytophthora capsici* strain LT263 was revived on 10% V8 agar medium (10% V8 juice, 0.14% CaCO_3_, 1.5% agar) at 25 °C in the dark for 3–5 days. Infiltrated leaves were detached and placed in humid chambers (>95% relative humidity) at 16 h post-agroinfiltration. Mycelial plugs (0.5 cm diameter) were excised from the margins of actively growing colonies and placed onto the abaxial surface of infiltrated regions. Inoculated leaves were then incubated at 25 °C for 48 h. Disease symptoms were documented under UV illumination using a Nikon digital camera (Nikon, Tokyo, Japan). Lesion areas were quantified using ImageJ 1.51j8 by manually outlining necrotic regions and calculating pixel areas.

*Phytophthora infestans* strain 1306 was cultured on rye agar medium at 18 °C in the dark for 14 days. Zoospores were released by incubating sporangia in cold water at 4 °C for 2 h, followed by filtration to remove mycelial debris. A 10-μL aliquot of zoospore suspensions (1.0 × 10^5^ zoospores/mL) was inoculated onto the abaxial leaf surface, followed by incubation of the inoculants at 18 °C and 100% relative humidity for 5 days. Disease lesion areas were quantified and statistically analyzed as described in 4.10 Statistical Analysis.

### 4.7. Trypan Blue Staining

Cell death was visualized using trypan blue staining. Infiltrated leaves were submerged in the staining solution (0.4% Trypan Blue, 25% lactic acid, 25% glycerol, 50% phenol) and boiled for 10 min. The leaves were subsequently incubated overnight at 37 °C to ensure complete staining. The leaves were then destained in absolute ethanol at 37 °C, with several ethanol changes, until chlorophyll was removed. Clear tissues were stored in 50% glycerol and photographed using a digital camera.

### 4.8. Phylogenetic Analysis

Phylogenetic analysis was performed using MEGA 11.0.13 software [23]. An unrooted maximum-likelihood phylogenetic tree was constructed using RDA2 homologous proteins from *A. thaliana*, *N. benthamiana*, and other species of Solanaceae (identified by BLAST 2.12.0+). Bootstrap support values (1000 replicates) were highlighted next to the branches to represent the percentage of replicate trees in which the corresponding taxa formed a monophyletic cluster. The tree was drawn to scale, with branch lengths proportional to the evolutionary distances used for tree inference. Evolutionary distances were calculated using the Poisson correction method and expressed as the number of amino acid substitutions per site. Protein sequences of RDA2 and its homologs were extracted from relevant studies on *N. benthamiana* [24], *A. thaliana* [25], *S. lycopersicum* cv. MicroTOM [26], and *S. tuberosum* cv. A157 (accession A6-26) [22]. Protein sequences used for phylogenetic analysis are detailed in Supplementary Text S2.

### 4.9. Quantitative Real-Time PCR Analysis

Total RNA was extracted using the Qiagen Plant RNA Kit (Qiagen Bio-Tek, Hilden, Germany) following the manufacturer‘s instructions. First-strand cDNA was synthesized from 1 μg of total RNA using HiScript III RT SuperMix (Vazyme, Nanjing, China) in a final volume of 20 μL. Then, quantitative real-time PCR (qRT-PCR) was performed using SYBR Green chemistry on a QuantStudio 5 Real-Time PCR System (Applied Biosystems, Foster City, CA, USA). Each reaction was conducted in a total volume of 10 μL, comprising 5 μL of 2 × SYBR Green Master Mix (Takara Bio, Shiga, Japan), 0.2 μL of forward and reverse primer (10 μM), 1 μL of diluted cDNA (1:10), and 3.2 μL of RNase-free water. The thermal cycling conditions were as follows: an initial denaturation at 95 °C for 30 s, followed by 40 cycles of denaturation at 95 °C for 5 s and annealing at 60 °C for 30 s. Melt curve analysis was performed between 60 °C and 95 °C to verify the specificity of the amplified products.

All reactions were performed in two technical replicates and three biological replicates. The potato *EF1α* gene was used as an internal reference for transcript level normalization. The relative gene expression levels were calculated using the 2^−ΔΔCt^ method. Statistical significance was determined by one-way analysis of variance (ANOVA) using OriginPro 2025 software (OriginLab, Northampton, MA, USA).

### 4.10. Statistical Analysis

All experiments were performed with three independent biological replicates. Statistical analyses were conducted using OriginPro 2025 (OriginLab). One-way analysis of variance (ANOVA) followed by Tukey’s multiple comparison test was applied for comparisons involving more than two groups. Fisher’s exact test was applied to analyze the suppression of StRDA2A-induced cell death by candidate RXLR effectors. In contrast, a two-tailed unpaired Student’s *t*-test was used for pairwise comparisons. The level of statistical significance was evaluated at a threshold of *p* < 0.05.

## Figures and Tables

**Table 1:** Quantitative analysis of StRDA2A-induced cell death suppression by candidate RXLR effectors.

Lab Code	Gene ID	Description	Dead Leaf Zones/All Leaf Zones	Inhibition Ratio (%)	Fisher’s Exact Test
GFP	-	Negative control	0/92	0	
CE08	PITG_05750	Attenuates the flg22-induced FRK1 up-regulation in *Arabidopsis* [13]	42/92	45.7	*p* < 0.001
CE47	PITG_04196	Inhibits cell death caused by INF1, BAX, and NIP [14]	10/92	10.9	*p* = 0.002
CE49	PITG_06308	Inhibits cell death caused by INF1, BAX, and NIP; ADP-ribose/NADH pyrophosphatases [14,15]	62/89	69.7	*p* < 0.001
GR040	PITG_15679	ADP-ribose/NADH pyrophosphatases [15]	14/46	30.4	*p* < 0.001
CE52	PITG_09160	Inhibits cell death caused by INF1, BAX, and NIP [14]	21/90	23.3	*p* < 0.001

## Data Availability

No new datasets were generated during the study.

## Supplementary Materials

The following supporting information can be downloaded at https://www.mdpi.com/article/10.3390/plants15060906/s1. Supplementary Figure S1. Characterization of four RDA2 family genes in the diploid potato inbred line A157. Supplementary Figure S2. Transient expression of AtRDA2 and StRDA2 homologs induces cell death in transgenic *Nicotiana benthamiana* expressing *NahG*. Supplementary Figure S3. Conserved K residue in ATP binding pocket of kinase domains of multiple RLKs. Supplementary Table S1. Primers used in this study. Supplementary Text S1. Coding sequences of StRDA2 proteins. Supplementary Text S2. RDA2 protein sequences for phylogenetic analysis. Supplementary Text S3. Gene sequences of StRDA2-D.

## Abbreviations

The following abbreviations are used in this manuscript:

PRRs	pattern recognition receptors
PAMPs	pathogen-associated molecular patterns
PTI	pattern-triggered immunity
NLR	nucleotide-binding leucine-rich repeat
ETI	effector-triggered immunity
ROS	reactive oxygen species
MAPK	mitogen-activated protein kinase
LecRLKs	lectin receptor-like kinases
LORE	lipooligosaccharide-specific reduced elicitation
RDA2	resistant to DFPM-inhibition of abscisic acid signaling 2
ABA	abscisic acid

## References

[1] Jones J.D.G., Staskawicz B.J., Dangl J.L. (2024). The plant immune system: From discovery to deployment. Cell.

[2] Zipfel C. (2014). Plant pattern-recognition receptors. Trends Immunol..

[3] Sun Y.L., Qiao Z.Z., Muchero W., Chen J.G. (2020). Lectin receptor-like kinases: The sensor and mediator at the plant cell surface. Front. Plant Sci..

[4] Luo X.M., Wu W., Liang Y.B., Xu N., Wang Z.Y., Zou H.S., Liu J. (2020). Tyrosine phosphorylation of the lectin receptor-like kinase LORE regulates plant immunity. Embo J..

[5] Liu L., Liu J., Xu N. (2023). Ligand recognition and signal transduction by lectin receptor-like kinases in plant immunity. Front. Plant Sci..

[6] Kato H., Nemoto K., Shimizu M., Abe A., Asai S., Ishihama N., Matsuoka S., Daimon T., Ojika M., Kawakita K. (2022). Recognition of pathogen-derived sphingolipids in *Arabidopsis*. Science.

[7] Park J., Kim T.H., Takahashi Y., Schwab R., Dressano K., Stephan A.B., Ceciliato P.H.O., Ramirez E., Garin V., Huffaker A. (2019). Chemical genetic identification of a lectin receptor kinase that transduces immune responses and interferes with abscisic acid signaling. Plant J..

[8] Wen Q., Wang S., Zhang X., Zhou Z. (2024). Recent advances of NLR receptors in vegetable disease resistance. Plant Sci..

[9] Haas B.J., Kamoun S., Zody M.C., Jiang R.H.Y., Handsaker R.E., Cano L.M., Grabherr M., Kodira C.D., Raffaele S., Torto-Alalibo T. (2009). Genome sequence and analysis of the Irish potato famine pathogen *Phytophthora infestans*. Nature.

[10] Senchou V., Weide R., Carrasco A., Bouyssou H., Pont-Lezica R., Govers F., Canut H. (2004). High affinity recognition of a *Phytophthora* protein by *Arabidopsis* via an RGD motif. Cell. Mol. Life Sci..

[11] Liu Y.N., Xu L., Wu M.S., Wang J.J., Qiu D., Lan J.M., Lu J.X., Zhang Y., Li X., Zhang Y.L. (2025). Three-step biosynthesis of salicylic acid from benzoyl-CoA in plants. Nature.

[12] Wang Q.Q., Han C.Z., Ferreira A.O., Yu X.L., Ye W.W., Tripathy S., Kale S.D., Gu B.A., Sheng Y.T., Sui Y.Y. (2011). Transcriptional programming and functional interactions within the *Phytophthora sojae* RXLR effector repertoire. Plant Cell.

[13] Zheng X.Z., McLellan H., Fraiture M., Liu X.Y., Boevink P.C., Gilroy E.M., Chen Y., Kandel K., Sessa G., Birch P.R.J. (2014). Functionally redundant RXLR effectors from *Phytophthora infestans* act at different steps to suppress early flg22-Triggered Immunity. PLoS Pathog..

[14] Yin J.L., Gu B., Huang G.Y., Tian Y., Quan J.L., Lindqvist-Kreuze H., Shan W.X. (2017). Conserved RXLR effector genes of *Phytophthora infestans* expressed at the early stage of potato infection are suppressive to host defense. Front. Plant Sci..

[15] Dong S.M., Yin W.X., Kong G.H., Yang X.Y., Qutob D., Chen Q.H., Kale S.D., Sui Y.Y., Zhang Z.G., Dou D.L. (2011). *Phytophthora sojae* avirulence effector Avr3b is a secreted NADH and ADP-ribose pyrophosphorylase that modulates plant immunity. PLoS Pathog..

[16] Tang D.Z., Wang G.X., Zhou J.M. (2017). Receptor kinases in plant-pathogen interactions: More than pattern recognition. Plant Cell.

[17] Kong L., Rodrigues B., Kim J.H., He P., Shan L.B. (2021). More than an on-and-off switch: Post-translational modifications of plant pattern recognition receptor complexes. Curr. Opin. Plant Biol..

[18] Bigeard J., Colcombet J., Hirt H. (2015). Signaling mechanisms in pattern-triggered immunity (PTI). Mol. Plant.

[19] Wang C., Wang G., Zhang C., Zhu P.K., Dai H.L., Yu N., He Z.H., Xu L., Wang E.T. (2017). OsCERK1-mediated chitin perception and immune signaling requires receptor-like cytoplasmic kinase 185 to activate an MAPK cascade in Rice. Mol. Plan.

[20] Yu X.Q., Niu H.Q., Liu C., Wang H.L., Yin W.L., Xia X.L. (2024). PTI-ETI synergistic signal mechanisms in plant immunity. Plant Biotechnol. J..

[21] Ren Y.J., Armstrong M., Qi Y.T., McLellan H., Zhong C., Du B.W., Birch P.R.J., Tian Z.D. (2019). *Phytophthora infestans* RXLR effectors target parallel steps in an immune signal transduction pathway. Plant Physiol..

[22] Zhang C.Z., Yang Z.M., Tang D., Zhu Y.H., Wang P., Li D.W., Zhu G.T., Xiong X.Y., Shang Y., Li C.H. (2021). Genome design of hybrid potato. Cell.

[23] Tamura K., Stecher G., Kumar S. (2021). MEGA11: Molecular evolutionary genetics analysis version 11. Mol. Biol. Evol..

[24] Kurotani K.I., Hirakawa H., Shirasawa K., Tanizawa Y., Nakamura Y., Isobe S., Notaguchi M. (2023). Genome sequence and analysis of *Nicotiana benthamiana*, the model plant for interactions between organisms. Plant Cell Physiol..

[25] Reiser L., Bakker E., Subramaniam S., Chen X.G., Sawant S., Khosa K., Prithvi T., Berardini T.Z. (2024). The *Arabidopsis* information resource in 2024. Genetics.

[26] Xue J.Y., Fan H.Y., Zeng Z., Zhou Y.H., Hu S.Y., Li S.X., Cheng Y.J., Meng X.R., Chen F., Shao Z.Q. (2023). Comprehensive regulatory networks for tomato organ development based on the genome and RNAome of MicroTom tomato. Hortic. Res..

