# Supplementary material for: Identification and Functional Analysis of Potato Receptor Kinase RDA2 Proteins
Source: Plants (Basel). 2026 Mar 14;15(6):906. doi: 10.3390/plants15060906 (PMC13030833; doi:10.3390/plants15060906)
Supplement: Supplementary file 1 [file plants-15-00906-s001.zip › plants-4142743-supplementary.pdf]

Supplementary Figures

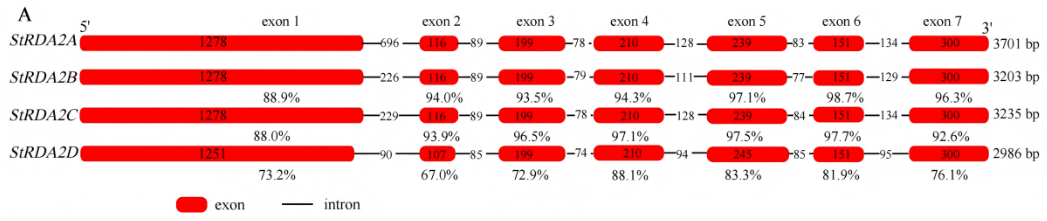

B

	StRDA2A	*StRDA2B*	*StRDA2C*	*StRDA2D*
StRDA2A	100	--	--	--
StRDA2B	92.2	100	--	--
StRDA2C	91.6	93.2	100	--
StRDA2D	76.9	76.4	76.4	100

C

	AtRDA2	StRDA2A	StRDA2B	StRDA2C	StRDA2D
AtRDA2	100	--	--	--	--
StRDA2A	50.6	100	--	--	--
StRDA2B	49.5	88.6	100	--	--
StRDA2C	51	85.8	89.2	100	--
StRDA2D	52.4	65.2	64.5	64.4	100

Supplementary Figure S1. Characterization of four *RDA2* family genes in the diploid potato inbred line A157.

(A) Exon-intron distribution of four *StRDA2* genes. Numbers in exons and introns indicate nucleotide lengths (unit, bp). Percentages represent the sequence identity of each exon in *StRDA2B*, *StRDA2C*, *StRDA2D* relative to the corresponding exon in *StRDA2A*, with values retained to one decimal place. Gene sequences were included in Supplementary Text 3. **(B)** Percent identity matrix of CDS sequences of four *StRDA2* genes. DNA sequences are provided in Supplementary Text 1. **(C)** Amino acid identity matrix of StRDA2A-D and AtRDA2 proteins. Protein sequences are provided in Supplementary Text 2.

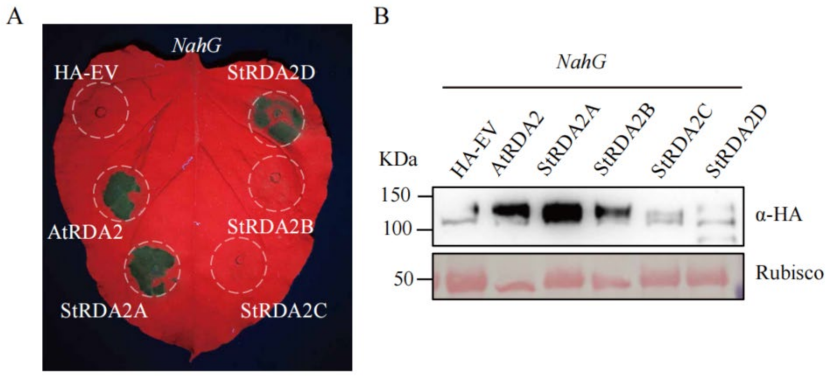

Supplementary Figure S2. Transient expression of *AtRDA2* and *StRDA2* homologs induces cell death in transgenic *N. benthamiana* expressing *NahG*.

(**A**) Cell death phenotypes in *transgenic N. benthamiana* expressing *NahG (Nb NahG)* at 48 post-infiltration (hpi). **(B)** Protein accumulation of AtRDA2 and StRDA2 proteins in leaves of *N. benthamiana NahG* at 24 hpi.

```
AtFLS2_Q9FL28                       870 FNSANIIGSSSLSTVYKGQL-EDGTVIAVKVLNLKEFSA
AtEFR_C0LGT6                        712
FSSTNLIGSGNFGNVFKGLLGPENKLVAVKVLNLL--KH
AtBRI1_O22476                       883 FHNDSLIGSGGFGDVYKAIL-KDGSAVAIKKLIHV--SG
AtRDA2_Q9SXB8                       524 FSLRNKLGQGGFGPVYKGKL-PEGQEIAVKRLSRK--SG
A157_02G016820.2_(StRDA2D)          503 FHLSSKLGQGGFGPVYKGKL-PDGQEIAVKRLSQS--SG
A157_02G016830.1_(StRDA2B)          515 FHSASKLGQGGFGPVYKGKL-PDGQEIAVKRLSQS--SG
A157_02G016860.1_(StRDA2C)          515 FQSASKLGEGGFGPVYKGKL-PDGQEIAVKRLSQF--SG
A157_02G016870.2_(StRDA2A)          515 FHPASKLGQGGFGPVYKGQL-PDGQEIAVKRLSQS--SG
```

Supplementary Figure S3. Conserved K residue in the ATP binding pocket of kinase domains of multiple RLKs.

Sequences of AtFLS2, AtEFR, AtBRI1 and AtRDA2 were downloaded from the Uniprot database. Protein sequence alignment of ATP binding pocket region was shown. Red font K indicates the predicted ATP binding site.

Supplementary Table

Supplementary Table S1. Primer information used in this study.

Primer name	Sequence (5′-3′)
pCAMBIA1305-AtRDA2-assbF	TATTTACAATTACggtaccATGGTGGTTTCAGTGACCATAC
pCAMBIA1305-AtRDA2-assbR	GGAACATCGTATGGGTAaagcttACGTCCTGTTACAGCTGTGA
pCAMBIA1305-StRDA2A-assbF	TATTTACAATTACggtaccATGAGCATATCTATGCTTTTTCATCT
pCAMBIA1305-StRDA2A-assbR	GAACATCGTATGGGTAaagcttTCGTGGTTCCAATATAGTAATG
pCAMBIA1305-StRDA2B-assbF	TTACTATTTACAATTACGGTACCcagtATGAGCTTATATCGATCATTTCT
pCAMBIA1305-StRDA2B-assbR	GGAACATCGTATGGGTAaagcttTCGTGGTTCCAATACAGTTATGG
pCAMBIA1305-StRDA2C-assbF	TTACTATTTACAATTACGGTACCCAGTATGAGCTTATATCGATCATTTCT
pCAMBIA1305-StRDA2C-assbR	GGAACATCGTATGGGTAaagcttTCGTGGTTCCAATACAGTTATGG
pCAMBIA1305-StRDA2D-assbF	ATTTACAATTACggtaccATGAAATTAAGCACAAGAGAGATACT
pCAMBIA1305-StRDA2D-assbR	AACATCGTATGGGTAaagcttTCGTCCTTCCATAATAGTAACG
pCAMBIA1305-A157StRDA2A^{K543M}-F	ATTGCAGTAATGAGGCTTTCACAGTCTTCTGG
pCAMBIA1305-A157StRDA2A^{K543M}-R	GAAAGCCTCATTACTGCAATTTCTTGTCCATCTG
StRDA2A-RTF	GCAGAAGATAGGCCAAATGTC
StRDA2A-RTR	ACGGACCCTTGGCTTTTAGA
StRDA2B-RTF	TCAAGCTTCGAAAAAGAGGTTG
StRDA2B-RTR	TGTGGTGAAGCATGTCCTCT
StRDA2C-RTF	GGCAATCAAGATCAGGCCAA
StRDA2C-RTR	TCGTGGTTCCAATACAGTTATGG
StRDA2D-RTF	CCAAGATAACTGACCTGCAC
StRDA2D-RTR	CATTGTTAATGGAGCCTTGAGA
StEF1A-RTF	CTGCTGCAACAAGATGGATG
StEF1A-RTR	CTGAAGTGGGAGACGGAGT
pMDC-CE08-assbF	CGGCGGTGGCGGTAGCactagtCGCTCGGCCACCGAACATG
pMDC-CE08-assbR	ATCGGGGAAATTCgagctcCTACGTAGCAGCAGTCTTGATCT
pMDC-CE47-assbF	CGGCGGTGGCGGTAGCactagtTCCATTCGCACCCCTGAAA
pMDC-CE47-assbR	ATCGGGGAAATTCgagctcCTATGTGTAAGACTTACTTCGCTGA
pMDC-CE49-assbF	CGGCGGTGGCGGTAGCactagtTCGATCTCTTCTTTCTCCGACC
pMDC-CE49-assbR	ATCGGGGAAATTCgagctcCTAAAGAGCACGACATACTCCTCC
pMDC-GR040-assbF	GGCGGTGGCGGTAGCaTCCCAAGCGGTCTTGCGAAG
pMDC-GR040-assbR	CGGGGAAATTCgagctcAACGTACTCCTCCTTCTTCGA
pMDC-CE52-assbF	CGGCGGTGGCGGTAGCactagtTCTCCCGGTGCGGACGCT
pMDC-CE52-assbR	ATCGGGGAAATTCgagctcCTAAGCCTTGTTGTTTTGTTCCTTCT

Restriction enzyme sites (lowercase) and homologous recombination arms (underlined) indicated.

Supplementary Text S1. Coding sequences of StRDA2 proteins

>A157_02G016870.2 (*StRDA2A*) CDS
ATGAGCATATCTATGCTTTTTCATCTTTTACTCTATTGTTTTTATGTAGTTTTTTCCAGTGCCAATACTTCAGACAC
CATTAGCAGTAGTGAGCCTGTGAGAGACTCAGAAACTGTATTTTCTAGAGGCAAAAGATTTAAACTGGGTTTT
TTTAGCCCCAGGAATTCTGCAAAACGTTATGTAGGGATTATGTTTAACCTACCATCACCAACACCAACTGTAGT
ATGGGTAGCTAACAGAGATGAGCCTTTACACGATTCTAGTGGAATACTCACAATATCAGAAGATAGCAATCTT
GTCATGTTGAATGGACAGAAGGAGATAATTTGGTCATCCAGTATTTCAAACTCTATGAAGAATTCCACAGCAC
AACTCTTGGACACTGGAAACTTAGTCTTGAAAGACAGCTCAAATGGGAGAGTTCTATGGGAAAGCTTTCAAT
ATCCTACGGATTCTCTCTTACAGCTCATGAAAATGGGCACTGATAAGCGTACTAACACAACAACTCTCCTGAA
CTCATGGGGGAGTCCTGATGATCCATCCGTTGGGAGCTTTTCAGCTGGAATTCAACATCGATACATTCCTCAGT
TTCTTATTTGGAAAAACAGTTCTCCTTACTGGCGGGGCGGTCCATGGAATAAACAGATATACATAGGATTACC
AGAAATGAATTCTTTCTATCTCTATGGTATTGACCTAGTAATTGATAATGCTGGCACAGCATACCAAACTTATTC
AGACCCAAATCAGTCTAGGATACTCTATTATTCCTTGAATTCAACAGGGTCTTACCAGGAGAAGGTTTGGGAT
CAAAGTAAGAAGGATTGGGTGGTAACATGGGCAAATCCCCGAACAGAGTGTGATTTTTATGCCAAGTGTGGG
ACATATGGAAGCTGTAATCCAATGAATTCTCCAATATGCAGTTGTATACAAGGTTTCAAACCAAAAAATGATG
GAGAATGGAAGAAGGGAGATTGGTCTGGTGGATGCATCAGAAGTACTGCACTAGACTGTGAAAGGAACAAA
ACTGATATTGAGAAGGACAAGCAGGACGGGTTTCTGAAGCTGCAGACAATGGGAGTGCCAGATTTTGTAATT
TCGGTACCTTCTGCAAAAAAAGACTGTGAGAGTGACTGTTTGAGTAACTGTTCCTGCACTGCATATTCATACT
ACAGAGGCATAGGTTGTATGCATTGGAATAGAAGCTTAGTTGATATTCAAGAATACTCCAGGGATGGGGCGGC
TAGTTTGTTCATTCGCCTTGCCTACTCTGAACTTGCTGCAAATGACAAGAAAGACATTCCTGTAGCAGCTATTG
CAACCACAGTCTCAATAGGCTCAATAACAGTTATCTTGTGTGGATATCTTTTCTGGAAATTGTTGGCTAAGCAC
AGAGAAAGGAAGAGGAAAAATGAAGCACTCTTAAGAGAAGCATCTCCAAAATTTTACCAAGAAGGCATGA
GTAAAGATGACATCAATCAAGTCAAAATTGAAGACATCACCTTGTACAGATTTGACATGTTAGCGAATGCCAC
TGACAGTTTTCACCCAGCTAGCAAGCTGGGGCAAGGAGGCTTTGGTCCAGTCTACAAAGGACAATTGCCAGA
TGGACAAGAAATTGCAGTAAAAAGGCTTTCACAGTCTTCTGGTCAGGGGCTACAGGAGTTTATGAATGAGGT
CGTTGTGATTTCTAGACTACAACATCGTAATCTTGTTAGACTCCTTGGCTGTTGCACAGAGAGAGGGGAAAAG
ATGCTGATTTATGATTTCATGCCAAATAGAAGCTTAGATGCATATCTTTTTGGTTCACACCAGGAAAAGTTCCTT
GATTGGAGTAAACGAGCCATCATTATTGAAGGAACTGGTCGAGGCCTCCTTTACCTTCACAGAGACTCAAGA
CTACGAATTATTCATAGAGATCTGAAGGCAAGCAATATCCTGTTGGATGAATACCTAAACCCAAAAATTTCGG
ATTTTGGCATGGCGAGGATTTTTGGAGGCAATCAAGATCAGGCCAGCACTATAAGAGTTGTTGGCACATATGG
TTACATGGCCCCTGAATATGCAATGTACGGAAGATTCTCAGAAAAATCAGATGTCTACAGCTTCGGTGTGTTG
ATATTGGAAATTGTCAGTGGAAGGAAGAACTCTAGTTTTTATGATGATGAAGATGAACTGACTCTACTTGCATA
TGCATGGAAGTTGTGGAATGAAAACAATATCATAAAATTGATAGACCCCAAAATATTTGATTCAAGCTTTGAA
AAACAGATGGTGAGATGTGGACATATTGGATTATTATGTGTTCAAGAATATGCAGAAGATAGGCCAAATGTCT
CCACAGTTCTGTCAATGCTCACTAGTGATATTGCTGAACTACCTACTCCTAAACAACCTGCATTTACCGGAGG
ACATGCTTCACCACAGCAAGTATCTTCTAAAAGCCAAGGGTCCGTGAATGCTGATACCATTACTATATTGGAA
CCACGATAG
>A157_02G016830.1__(StRDA2B) CDS
ATGAGCTTATATCGATCATTTCTTCTTTTGCTTTGTTGCTTTTATGTAGTTTTTTCTGGTGCCAATGCTTCAGACAC
CATTACCAGTAGTGAGCCCGTGAGGGACTCGGAAACTGTCTTTTCCAGTGGCAAAACATTTAAACTGGGATTT
TTCAGTCCTGGGAATTCTGCAAATCGTTATGTAGGGATTATGTTTAACCTACCATCACCAACACCAACTGCAGT
ATGGGTAGCTAACAGAGACAAGCCTATAAATGATTCTAGCGGATTACTCACACTATCAGAAGATGGCAATCTT
GTAATCTTGAATGGACAGAAGGAGATAATATGGTCATCCAATATTTCAAACTCTATGAAGAATTCTACTGCTCA
ACTCTTGGACACTGGCAACTTAGTCTTGAAAGACAGCTCAAACGGGAAAGTTCTATGGGAAAGCTTTCAATA
TCCTACAGATTCTGTCTTACAGCTCATGAAAATGGGCATTGATAAGAGTACTAACACAACGGCTCTCCTGAAA
TCATGGAGAAGTCCTGATGATCCATCTGTTGGGAGCTTCTCAGCTGGAATTCAACTTCAATACATTCCCCAGG
CTTTTATTTGGAATAACACCGTTCCTTACTGGCGTAGTAGTCCATGGGATAAACAGATCTATATTGGATTACCAG
AAATGAAATCTTCCTATCGCTCTGGTGTTGACCTTGTAGCTGATAATGCTGGCACCGCATACCAAACTTATTCC
AATGGAAATCAGTCTTGGATACTCTATTATTCCTTGAATTCAACAGGGTCTTATCAGGAGAAGGTTTGGGATCA
AAGTAAGAAGGATTGGGTGGTAACATGGGCAAATCCCCGAAGTGAGTGTGATTTTTATGCTAAGTGCGGGGC
ATTTAGTAGTTGTAATCCAAAGAGCTCTCCAATATGCAGTTGCATACAAGGTTTTAAGCCTAAAAATGAAGGA
GAATGGGAGAAAGGAGAATGGTCTGGTGGATGCATCAGAAGGACTGCATTAGACTGTGAAAGGAACAAAAC
TGATGTTGAGAAGGGCAAAAAGGATGGGTTTTTGAAGATGCAGACAATGGGAGTACCAGATTTTGTAATTTG
GGTATCCTCTGCGAAAGAAGACTGTGAAAGTGACTGTTTAAGTAACTGTTCCTGCATGGCATATTCATACTAC
ACAGGCATTGGTTGTATGCATTGGAATAGAAGCTTAATTGATATTCAAGAATACTACATGGATGGGGCGGCTG
ATTTGTTCATTCGTCTTGCCTACTCCGAACTTGCTGCAAATGACAAGAAAGACTTCCCTGTAGCAGCTATTGCA
ATCACAGTTTCGATAGGCTCAATAATAGTTATCTTATGTGGATATCTTTTCTGGAAATTGTTGGCTAAGCACAGA

GAAAGAAAGAGGAAAAAAGAAGCATTCTTAAGAGAAGCATCTTCAAAATTTTACCAAGGCGGCATGATTAA
AGATGACATCAATCAAGTCAAAATTGAAGATATCACCTTGTACAGCTTTGACATGTTAGCAAATGCAACTGAC
AGATTTCACTCAGCTAGCAAGCTGGGGCAAGGAGGCTTTGGTCCAGTCTACAAAGGAAAATTGCCAGATGGA
CAAGAAATTGCAGTAAAAAGGCTTTCACAGTCTTCTGGTCAGGGGGAACAGGAGTTTATGAATGAGGTGGTC
GTGATTTCTAGACTACAACATCATAATCTTGTTAGACTCCTCGGCTGCTGCACAGAAAGAGGGGAAAAGATGC
TGGTGTATGATTTCATGCCCAATAGAAGCTTAGATGCATATCTTTTTGGTTCACACCAGGAAAGGTTCCTTGATT
GGAGTAAACGAGCCATCATTATTGAAGGAACTGGTCGAGGCCTCCTTTACCTTCACAGAGACTCAAGACTAC
GAATTATTCATAGAGATCTGAAGGCAAGCAACATCCTGTTGGATGAATACCTAAACCCAAAAATTTCGGATTT
TGGCATGGCGAGGATTTTTGGAGGAAATCAAAACCAGGCACGCACTATAAGAGTTGTTGGCACATATGGTTA
CATGGCCCCTGAATATGCAATGCACGGAAGATTCTCAGAAAAATCAGATGTCTACAGCTTCGGTGTGTTGATC
TTGGAAATTGTCAGTGGAAGGAAGAACTCTAGTTTTTATGATGATGAAGATGAACTGACTCTACTTGCATATG
CATGGAAGTTGTGGAACGAAAACAATATCATAAAATTGATAGACCCCAAAATATTTGATTCAAGCTTCGAAA
AACAGATGGTGAGATGTGTACATACTGGATTATTATGTGTTCAAGAATATGCAGAAGATAGGCCAAATGTCTC
CACAGTTCTGTCAGTGCTCACGAGTGATATCGCTGAACTACCTACTCCTAAACAACCTGCATTTACCGGAGGG
CATGCTTCACCACAGCAAGGATCTTCTAAAAGCCAAGGGTCCGTGAATGCTGATACCATAACTGTATTGGAAC
CACGATAA
>A157_02G016860.1_(StRDA2C) CDS
ATGAGCTTATATCGATCATTTCTTGTTTTTCTCTGTTGCTTTTATGTAGTTTTTTCTGGTGCCAATGCTTCAGACAT
CATTACCAGTAGCGAACCCGTCAGGGACTCGGAAACTGTATTTTCCAGTGGTAAAAGATTTAAACTGGGATTT
TTCAGCCCAGGGAATTCTGCAAATCGTTATGTAGGGATTATGTTTAATCTACCATCACCAACACCAACTGCAGT
ATGGGTAGCTAACAGAGACAAGCCTATAAATGATTCTAGCGGAATATTCACAATATCAGATGATGGCAACCTT
GTAATCTTGAATGGACAGAAGGAGGTAATTTGGTCATCCAGTATTTCAAACTCCATGAAGAATTCCACTGCTC
AGCTCTTGGATACTGGCAACTTAGTCTTGAAAGACAGCTCAAATGGAAGAGTTATATGGGAAAGCTTTCAATA
TCCTACGGATTCTTTCTTACAGCTCATGAGAATGGGCATTTATAATAGTACTAACACAGCATCTCTCCTGAAAT
CATGGAGAAGTCCTGATGATCCATCTGTTGGGAGTTTCTCAGGTGGAATTCAACTTCAATACATCCCCCAGGC
TTTTATTTGGAATAACACCGCTCCTTACTTGCGTAGTAGTCCATGGAATAAACAGATCTATATTGGAATACCAG
AAATGATATCTTCCTACCGCTCTGGTTATGACCTCTTTGCTGATAATGTTGGCACCACATACCTAACTTATTCCC
ATGGAAATCAGTCTTTGATATTCTATTATTCCTTGAATTCAACAGGGTCTTATCAGGCGAAGGTTTGGGATCAA
AGTAAGAAGGATTGGGTGGTAACATGGGCAATTCCCCGCACTGAGTGTGATTTTTATGCTAAGTGCGGGACAT
TTGGAAGCTGTAATCCAAAGAATTCTCCAATATGCAGTTGTATACAAGGTTTTAAACCAAAACATGAAGGAG
AATGGGAGAAAGGAGATTGGTCTGGTGGATGCATCAGAACGACTGCACTACACTGTGAAAGGAACAGAAAT
GATGTTGAGAAGGGAAAGCAGGACGGGTTTTTGAAGCTGCAGACAATGGGAGTGCCAGATTTTGTAATTTCG
GCATCCTCAGCGAAAGAAGACTGTGAGAGTGACTGTTTGAATAACTGTTCCTGCACGGCATATTCATACTACT
CAGGCATAGGTTGTATGCATTGGAATAGAACTTTAATTGATATTCAACAATACTCCATGGATGGGGCGGCTGAT
TTGTTCATTCGCCTTGCCTACTCCGAACTTGCTGCAAATGAAAAGAAAAACGTTCCTGTAGCAGCTATTGCAA
TCACAGTCTCAATAGGCTCAATAACAGTTATCTTATGTGGATATCTTTTCAGGAAATTGTTGGCTAACCACAAA
GAAAGGAAGAGGAAAAATGAAGCACTCTTAAGAGAAGCATCTCCAAAATTTTACCAAGAAGGCATGATTAA
AGATGACATCAACCAAGTCAAAATTGAAGACATCACCTTGTACAGCTTTGACATGTTAGCAAATGCCACTGA
CAGTTTTCAATCAGCTAGCAAGCTGGGGGAAGGAGGCTTTGGTCCAGTCTACAAAGGGAAATTGCCAGATGG
ACAAGAAATTGCAGTAAAAAGGCTTTCACAGTTTTCTGGTCAGGGGCTACAGGAGTTTATGAATGAGGTCGT
CGTGATTTCTAGACTACAACATCGTAATCTTGTTAGACTCCTTGGCTGTTGCACAGAGAGAGGGGAAAAGATG
TTGGTTTATGATTTCATGCCAAATAGAAGCTTAGATACATATCTTTTTGGTTCACACCAGGAAAAGTTCCTTGAT
TGGAGTAAACGAGCTATCATTATTGAAGGAACTGGTCGAGGCCTCCTTTACCTTCACAGAGACTCAAGACTAC
GAATTATTCATAGAGATCTGAAGGCAAGCAACATCCTGTTGGATGAATACCTAAACCCAAAAATTTCGGATTT
TGGCACGGCGAGGATCTTTGGAGGCAATCAAGATCAGGCCAACACTATAAGAGTTGTTGGAACATATGGTTA
CATGGCCCCTGAATATGCAATGCATGGAAGATTCTCAGAAAAATCAGATGTCTACAGCTTCGGTGTGTTGTTAT
TGGAAATTGTTAGTGGAAGGAAGAACTCTAGTTTTTCTGATGATGAAGATGAACTGACTCTACTTGCATATGC
ATGGAAGTTGTGGAATAAAAACAATATTGTAAAATTGATAGACCCCAAAATATTTGATTCAAGCTTCGAAAAA
GAGGTTGTGAGATGTGTACATATTGGATTATTATGTGTTCAAGAATATGCAGAAGATAGGCCAAATGTCTCCAC
AGTTCTATCAATGATCACTAGTGATAACGCTGAACTACCTACTCCTAAACAACCTGCTTTTACCAGAGGACAT
GCTTCACCACAGCCAGGATCTTCTAAAAGAGAAGGTTCGGTGAATGCTGATACCATAACTGTATTGGAACCA
CGATAG
>A157_02G016820.2_ (StRDA2D) CDS
ATGAAATTAAGCACAAGAGAGATACTTCTGTTTCTTTCTATATCAACTGCTTCAGGTATCATTAGCACCAATAA
ATTCCTGCGAGATTCAGAAACTTTAGTCTCCAATGACAAAAGATTCATATTCGGGTTCTTTAGTCCTGAAAATT
CGACGAATCGTTATGTTGGTGTTATGTTTAATGTCCAACCACCAACTGTTGTATGGGTTGCCAACAGAGAGAA
ACCTTTACAGGATTCTAGTGGAAGAGTGACAATATCTGATGATGGAAATCTTGTAATCTTGAATTCACAGAATA
GGAGTATATGGTCATCAAATATTTCACCAGCTGTGAGAAATTCCACAGCGCAGATCTTGGATACTGGAAACTT

AGTTTTGAACGATAGCTTCAATGGGAGGGTTCTCTGGGAAAGTTTTCGGGATCCTTCAGATTGCTTCTTGCAG
ACCATGAAAATTGGCGTTGATGTAAGTACTAACACGACAAATCTGCTGAAATCATGGATAAGTCCTTCAGATC
CATCTGTTGGGAGTTTCTCAGTTGGTATTCAACCTGAAACAGTTCCCCAGATTTCCATATGGAAGAATGGGAA
ACCTCATTGGCGTAGTGGTCCATGGAATAAACAGGTTTTCATTGGGGTACCAGACATGACTTCATTCTATCTCA
ATGGATTTGATCTAGTTAATGACAACAAGGGCACCGTGTACCTTACCTATTTATATGCAAATCAGATTGAGCTG
ACGTTTTTCACCTTGAACTCAACAGGGTTTTTGCAGCAGAAATATATGGATCCTAGTAAGAATGATTGGGAAG
TAACATGGGAATTTCCTGCAACTGAGTGTGATTTTTATGGAAAATGTGGACCTTTTGGAAGCTGTGATCCTACA
AGCTCACCAATCTGTTCTTGTTTAGAGGGATTTAGGCCGACAAGTGAAGAGGAATGGGGAAAAGGAAACTG
GACTCGTGGATGCAACAGAAAGTCCATGTTAGAGAGCGAAAGAAACAGTTCTAACCTTGAGCAAGGGAAGC
AAGATTGGTTTCTGAAGCTGCAGTCAATGAAAGTGCCGGATTCTGCTATTTGGGTACCTTTTGCAGATGAAGA
TTGTGTTAACGGTTGCTTGAGGAATACTTCATGCATAGCTTATTCATACTACACAGGCATAGGATGCATGCATT
GGGAAGGAAGCTTACTTGATGTTCAGAAATTCTCCATCGGTGGGGCAGATTTATTCCTCCGCCTTTCATACTCT
GAGCGTGATCAAAAGAGAGAATATAAAGTAGTCATTGCCATCATAGTCCCAGTAGGCTCAATAATTCTTGCCA
TTTTCGGATACATTTCCTGCAAATATGTAGCTAAGCGCAGAGGATGGAAGAGAATGAGTAAGATCTTATCAAG
TGAATCATCGCCAAACTATTACAAGGAAGACAAGATTACAGAGGACATCAATCAAGCTAAATTGGAAGAACT
GCTTGTATACAACTTTGATATCTTAGCAAACGCAACTGAGAATTTTCATCTGTCCAGCAAGCTTGGACAGGGA
GGTTTTGGTCCAGTTTACAAAGGGAAATTGCCAGATGGACAAGAGATTGCTGTGAAAAGGCTTTCACAGTCT
TCTGGTCAGGGGCTGCAGGAGTTCATGAATGAGGTTGTGGTGATTTCAAAACTTCAACATCGTAATCTTGTTA
GACTTTTTGGGTGCTGCATAGAAAGAGGGGAAAAGATTCTGGTTTATGAATACATGCCAAAAAGAAGCTTGG
ATGCCTATCTCTTTGGGTCACAACAACAAGAGGAAGAGTTCCTGGATTGGAGTAAACGTGTGATCATTATTGA
GGGAATTGGTCGAGGCCTTCTTTACCTTCACAGGGATTCAAGACTAAGGATTATTCATAGGGATTTAAAGGCC
AGCAACATTTTGTTGGATGAATACCTGAACCCCAAAATTTCAGATTTTGGGATGGCAAGGATTTTTGCAGGCA
ACCAAGATCAGGCCAACACAAGCAGAGTAGTTGGAACCTATGGTTACATGGCACCTGAATATGCAATGGAAG
GAAGATTCTCAGAAAAATCAGATGTTTATAGCTTTGGAGTATTGTTATTGGAAATTATAAGTGGAAGGAGGAA
CACTAGCTTTCACCAAGATGATGGTGCATTAAGCCTGCTAGCATTGGCGTGGAAATGTTGGATTGGAAACAAG
ATTGTGGAATTGGTTGATCCCAAGATAACTGACCTGCACCTTGAAAAAGAAATGGTGAGATGTGTACAAGTT
GGATTATTATGTGTACAAGAATATGCAGAAGACAGACCAAATGTCTCCACAATTTTGTCTATGCTCACCAGGG
AAATTGATGATTTACCAAGTCCTAAACAACCTGCATTTACAACAAGACCGAGCTTTTCCAAAAAATGCACTTC
TAAATCTCAAGGCTCCATTAACAATGTTACCGTTACTATTATGGAAGGACGATAA

Supplementary Text S2. RDA2 protein sequences for phylogenetic analysis.

>AT1G11330.2_(AtRDA2)
MVVSVTIRRRFVLLLLACTCLLSRRLCFGEDRITFSSPIKDSESETLLCKSGIFRFGFFTPVNSTTRLRYVGIWYEKIPIQT
VVWVANKDSPINDTSGVISIYQDGNLAVTDGRNRLVWSTNVSVPVAPNATWVQLMDSGNLMLQDNRNNGEILWE
SFKHPYDSFMPRMTLGTDGRTGGNLKLTSWTSHDDPSTGNYTAGIAPFTFPELLIWKNNVPTWRSGPWNGQVFIGL
PNMDSLLFLDGFNLNSDNQGTISMSYANDSFMYHFNLDPEGIIYQKDWSTSMRTWRIGVKFPYTDCDAYGRCGRF
GSCHAGENPPCKCVKGFVPKNNTEWNGGNWSNGCMRKAPLQCERQRNVSNGGGGGKADGFLKLQKMKVPISA
ERSEASEQVCPKVCLDNCSCTAYAYDRGIGCMLWSGDLVDMQSFLGSGIDLFIRVAHSELKTHSNLAVMIAAPVIGV
MLIAAVCVLLACRKYKKRPAPAKDRSAELMFKRMEALTSDNESASNQIKLKELPLFEFQVLATSTDSFSLRNKLGQG
GFGPVYKGKLPEGQEIAVKRLSRKSGQGLEELMNEVVVISKLQHRNLVKLLGCCIEGEERMLVYEYMPKKSLDAYLF
DPMKQKILDWKTRFNIMEGICRGLLYLHRDSRLKIIHRDLKASNILLDENLNPKISDFGLARIFRANEDEANTRRVVG
TYGYMSPEYAMEGFFSEKSDVFSLGVIFLEIISGRRNSSSHKEENNLNLLAYAWKLWNDGEAASLADPAVFDKCFEKE
IEKCVHIGLLCVQEVANDRPNVSNVIWMLTTENMSLADPKQPAFIVRRGASEAESSDQSSQKVSINDVSLTAVTGR
>A157_02G016820.2_(StRDA2D)
MKLSTREILLFLSISTASGIISTNKFLRDSETLVSNDKRFIFGFFSPENSTNRYVGVMFNVQPPTVVWVANREKPLQDSS
GRVTISDDGNLVILNSQNRSIWSSNISPAVRNSTAQILDTGNLVLNDSFNGRVLWESFRDPSDCFLQTMKIGVDVSTN
TTNLLKSWISPSDPSVGSFSVGIQPETVPQISIWKNGKPHWRSGPWNKQVFIGVPDMTSFYLNGFDLVNDNKGTVYL
TYLYANQIELTFFTLNSTGFLQQKYMDPSKNDWEVTWEFPATECDFYGKCGPFGSCDPTSSPICSCLEGFRPTSEEEW
GKGNWTRGCNRKSMLESERNSSNLEQGKQDWFLKLQSMKVPDSAIWVPFADEDCVNGCLRNTSCIAYSYYTGIGC
MHWEGSLLDVQKFSIGGADLFLRLSYSERDQKREYKVVIAIIVPVGSIILAIFGYISCKYVAKRRGWKRMSKILSSESSP
NYYKEDKITEDINQAKLEELLVYNFDILANATENFHLSSKLGQGGFGPVYKGKLPDGQEIAVKRLSQSSGQGLQEFM
NEVVVISKLQHRNLVRLFGCCIERGEKILVYEYMPKRSLDAYLFGSQQQEEEFLDWSKRVIIIEGIGRGLLYLHRDSRLR
IIHRDLKASNILLDEYLNPKISDFGMARIFAGNQDQANTSRVVGTYGYMAPEYAMEGRFSEKSDVYSFGVLLLEIISG
RRNTSFHQDDGALSLLALAWKCWIGNKIVELVDPKITDLHLEKEMVRCVQVGLLCVQEYAEDRPNVSTILSMLTREI
DDLPSPKQPAFTTRPSFSKKCTSKSQGSINNVTVTIMEGR*
>A157_02G016830.1_(StRDA2B)
MSLYRSFLLLLCCFYVVFSGANASDTITSSEPVRDSETVFSSGKTFKLGFFSPGNSANRYVGIMFNLPSPTPTAVWVAN
RDKPINDSSGLLTLSEDGNLVILNGQKEIIWSSNISNSMKNSTAQLLDTGNLVLKDSSNGKVLWESFQYPTDSVLQLM
KMGIDKSTNTTALLKSWRSPDDPSVGSFSAGIQLQYIPQAFIWNNTVPYWRSSPWDKQIYIGLPEMKSSYRSGVDLV
ADNAGTAYQTYSNGNQSWILYYSLNSTGSYQEKVWDQSKKDWVVTWANPRSECDFYAKCGAFSSCNPKSSPICSCI
QGFKPKNEGEWEKGEWSGGCIRRTALDCERNKTDVEKGKKDGFLKMQTMGVPDFVIWVSSAKEDCESDCLSNCS
CMAYSYYTGIGCMHWNRSLIDIQEYYMDGAADLFIRLAYSELAANDKKDFPVAAIAITVSIGSIIVILCGYLFWKLLA
KHRERKRKKEAFLREASSKFYQGGMIKDDINQVKIEDITLYSFDMLANATDRFHSASKLGQGGFGPVYKGKLPDGQ
EIAVKRLSQSSGQGEQEFMNEVVVISRLQHHNLVRLLGCCTERGEKMLVYDFMPNRSLDAYLFGSHQERFLDWSKR
AIIIEGTGRGLLYLHRDSRLRIIHRDLKASNILLDEYLNPKISDFGMARIFGGNQNQARTIRVVGTYGYMAPEYAMHG
RFSEKSDVYSFGVLILEIVSGRKNSSFYDDEDELTLLAYAWKLWNENNIIKLIDPKIFDSSFEKQMVRCVHTGLLCVQE
YAEDRPNVSTVLSVLTSDIAELPTPKQPAFTGGHASPQQGSSKSQGSVNADTITVLEPR*
>A157_02G016860.1_ (StRDA2C)
MSLYRSFLVFLCCFYVVFSGANASDIITSSEPVRDSETVFSSGKRFKLGFFSPGNSANRYVGIMFNLPSPTPTAVWVANR
DKPINDSSGIFTISDDGNLVILNGQKEVIWSSSISNSMKNSTAQLLDTGNLVLKDSSNGRVIWESFQYPTDSFLQLMR
MGIYNSTNTASLLKSWRSPDDPSVGSFSGGIQLQYIPQAFIWNNTAPYLRSSPWNKQIYIGIPEMISSYRSGYDLFADN
VGTTYLTYSHGNQSLIFYYSLNSTGSYQAKVWDQSKKDWVVTWAIPRTECDFYAKCGTFGSCNPKNSPICSCIQGFK
PKHEGEWEKGDWSGGCIRTTALHCERNRNDVEKGKQDGFLKLQTMGVPDFVISASSAKEDCESDCLNNCSCTAYS
YYSGIGCMHWNRTLIDIQQYSMDGAADLFIRLAYSELAANEKKNVPVAAIAITVSIGSITVILCGYLFRKLLANHKER
KRKNEALLREASPKFYQEGMIKDDINQVKIEDITLYSFDMLANATDSFQSASKLGEGGFGPVYKGKLPDGQEIAVKR
LSQFSGQGLQEFMNEVVVISRLQHRNLVRLLGCCTERGEKMLVYDFMPNRSLDTYLFGSHQEKFLDWSKRAIIIEGT
GRGLLYLHRDSRLRIIHRDLKASNILLDEYLNPKISDFGTARIFGGNQDQANTIRVVGTYGYMAPEYAMHGRFSEKS
DVYSFGVLLLEIVSGRKNSSFSDDEDELTLLAYAWKLWNKNNIVKLIDPKIFDSSFEKEVVRCVHIGLLCVQEYAEDRP
NVSTVLSMITSDNAELPTPKQPAFTRGHASPQPGSSKREGSVNADTITVLEPR*
>A157_02G016870.2_(StRDA2A)
MSISMLFHLLLYCFYVVFSSANTSDTISSSEPVRDSETVFSRGKRFKLGFFSPRNSAKRYVGIMFNLPSPTPTVVWVANR
DEPLHDSSGILTISEDSNLVMLNGQKEIIWSSSISNSMKNSTAQLLDTGNLVLKDSSNGRVLWESFQYPTDSLLQLMK
MGTDKRTNTTTLLNSWGSPDDPSVGSFSAGIQHRYIPQFLIWKNSSPYWRGGPWNKQIYIGLPEMNSFYLYGIDLVI
DNAGTAYQTYSDPNQSRILYYSLNSTGSYQEKVWDQSKKDWVVTWANPRTECDFYAKCGTYGSCNPMNSPICSCIQ
GFKPKNDGEWKKGDWSGGCIRSTALDCERNKTDIEKDKQDGFLKLQTMGVPDFVISVPSAKKDCESDCLSNCSCT
AYSYYRGIGCMHWNRSLVDIQEYSRDGAASLFIRLAYSELAANDKKDIPVAAIATTVSIGSITVILCGYLFWKLLAKHR
ERKRKNEALLREASPKFYQEGMSKDDINQVKIEDITLYRFDMLANATDSFHPASKLGQGGFGPVYKGQLPDGQEIA

VKRLSQSSGQGLQEFMNEVVVISRLQHRNLVRLLGCCTERGEKMLIYDFMPNRSLDAYLFGSHQEKFLDWSKRAIII
EGTGRGLLYLHRDSRLRIIHRDLKASNILLDEYLNPKISDFGMARIFGGNQDQASTIRVVGTYGYMAPEYAMYGRFSE
KSDVYSFGVLILEIVSGRKNSSFYDDEDELTLLAYAWKLWNENNIIKLIDPKIFDSSFEKQMVRCGHIGLLCVQEYAED
RPNVSTVLSMLTSDIAELPTPKQPAFTGGHASPQQVSSKSQGSVNADTITILEPR*
>A157_10G000470.1
MASCIIISNWYIFLFSVFYINLLRCTCTSKISQGEILRDGDMLISPKRKFNLGFFSPNVSNNQRFVGIWYVDGPKNSFV
WVANRDKPIFDKNGVFTIEKNGNLVVKNGRGDLMWTSNVAAMNNNCTAHLSDYGNLMMFNGNNRELWQSFH
HPTDTFLPEMRFYLDEVLRSWTSESDPSPGRYSLGVNTRGSPQIVIFDGEKRRWRSGYWDGRIFTGVTDMKPEYLHG
FKLYNEGDKLYFTYTVSDPSDLVRFHISPTGYELEQRWDKDNENWSIIQSHPSGDCDLYNLCGNFAKCDITYLKKCIC
LVGFVPKDLGQWNARNWSEGCVRRKEVECRGNNSVLKSGSGKKDGFFEIEKIKLPDFADTAYLQNIDECRSMCLEN
CSCTAYAFVSGINCMMWSGDLVDMQQFQEGGNTLYVRLADSEFAGSNKTVKIVVISVMVAGAFLVCMVVFLLCKY
KAKTRVSNKINQMETSVPTRSGEFSMNTSGAGDLSIEGHQGSGSELIFFSFSGVAAATDNFSNENKLGQGGFGPVYK
GKLLCGVEIAVKRLSRKSGQGVEEFKNEIKLIAKLQHRNLVRLMGCCIEGEEKMLFYEYMANRSLDSFLFDPVKQAQ
LDWRKRFNIIEGIARGLLYLHRDSRLRIIHRDLKASNILLDEEMNPKISDFGMARIFGGNENEANTNRVVGTYGYMA
PEYAMEGLFSGKSDVYSFGVLLLEIICGRRNTSYRSNEHSGIIGYVCPITTSRYLL*
>AT1G11350.1
MGCLLILLLTLICFSLRLCLATDVITFSSEFRDSETVVSNHSTFRFGFFSPVNSTGRYAGIWFNNIPVQTVVWVANSNSP
INDSSGMVSISKEGNLVVMDGRGQVHWSTNVLVPVAANTFYARLLNTGNLVLLGTTNTGDEILWESFEHPQNIYLP
TMSLATDTKTGRSLKLRSWKSPFDPSPGRYSAGLIPLPFPELVVWKDDLLMWRSGPWNGQYFIGLPNMDYRINLFEL
TLSSDNRGSVSMSYAGNTLLYHFLLDSEGSVFQRDWNVAIQEWKTWLKVPSTKCDTYATCGQFASCRFNPGSTPPC
MCIRGFKPQSYAEWNNGNWTQGCVRKAPLQCESRDNNDGSRKSDGFVRVQKMKVPHNPQRSGANEQDCPESCL
KNCSCTAYSFDRGIGCLLWSGNLMDMQEFSGTGVVFYIRLADSEFKKRTNRSIVITVTLLVGAFLFAGTVVLALWKIA
KHREKNRNTRLLNERMEALSSNDVGAILVNQYKLKELPLFEFQVLAVATNNFSITNKLGQGGFGAVYKGRLQEGLD
IAVKRLSRTSGQGVEEFVNEVVVISKLQHRNLVRLLGFCIEGEERMLVYEFMPENCLDAYLFDPVKQRLLDWKTRFN
IIDGICRGLMYLHRDSRLKIIHRDLKASNILLDENLNPKISDFGLARIFQGNEDEVSTVRVVGTYGYMAPEYAMGGLF
SEKSDVFSLGVILLEIVSGRRNSSFYNDGQNPNLSAYAWKLWNTGEDIALVDPVIFEECFENEIRRCVHVGLLCVQDH
ANDRPSVATVIWMLSSENSNLPEPKQPAFIPRRGTSEVESSGQSDPRASINNVSLTKITGR*
>AT1G61390.1
MYKLPQRNCADKQEYTVHMRKMGMVIFACLLLLIIFPTFGYADINTSSPLSIGQTLSSPDGVYELGFFSPNNSRKQYV
GIWFKNIAPQVVVWVANRDKPVTKTAANLTISSNGSLILLDGTQDVIWSTGEAFTSNKCHAELLDTGNLVVIDDVS
GKTLWKSFENLGNTMLPQSSVMYDIPRGKNRVLTSWRSNSDPSPGEFTLEFTPQVPPQGLIRRGSSPYWRSGPWAKT
RFSGIPGIDASYVSPFTVLQDVAKGTASFSYSMLRNYKLSYVTLTSEGKMKILWNDGKSWKLHFEAPTSSCDLYRACG
PFGLCVRSRNPKCICLKGFVPKSDDEWKKGNWTSGCVRRTQLSCHTNSSTKTQGKETDSFYHMTRVKTPDLYQLA
GFLNAEQCYQDCLGNCSCTAFAYISGIGCLVWNRELVDTVQFLSDGESLSLRLASSELAGSNRTKIILGTTVSLSIFVIL
VFAAYKSWRYRTKQNEPNPMFIHSSQDAWAKDMEPQDVSGVNLFDMHTIRTATNNFSSSNKLGQGGFGPVYKGKL
VDGKEIAVKRLSSSSGQGTDEFMNEIRLISKLQHKNLVRLLGCCIKGEEKLLIYEYLVNKSLDVFLFDSTLKFEIDWQK
RFNIIQGVARGLLYLHRDSRLRVIHRDLKVSNILLDEKMIPKISDFGLARMSQGTQYQDNTRRVVGTLGYMAPEYAW
TGVFSEKSDIYSFGVLLLEIIIGEKISRFSEEGKTLLAYAWESWCETKGVDLLDQALADSSHPAEVGRCVQIGLLCVQH
QPADRPNTLELMSMLTTISELPSPKQPTFTVHSRDDDSTSNDLITVNEITQSVIQGR*
>AT1G61400.1
MDFLFLLLERKNKHMGKKRVVLLWLSIFISFSSAEITEESPLSIGQTLSSSNGVYELGFFSFNNSQNQYVGISFKGIIPRV
VVWVANREKPVTDSAANLVISSNGSLQLFNGKHGVVWSSGKALASNGSRVELLDSGNLVVIEKVSGRTLWESFEHL
GDTLLPHSTIMYNVHTGEKRGLTSWKSYTDPSPGDFVVLITPQVPSQGFLMRGSTPYFRSGPWAKTKFTGLPQMDES
YTSPFSLTQDVNGSGYYSYFDRDNKRSRIRLTPDGSMKALRYNGMDWDTTYEGPANSCDIYGVCGPFGFCVISVPPK
CKCFKGFIPKSIEEWKTGNWTSGCVRRSELHCQGNSTGKDANVFHTVPNIKPPDFYEYADSVDAEECQQNCLNNC
SCLAFAYIPGIGCLMWSKDLMDTVQFAAGGELLSIRLARSELDVNKRKKTIIAITVSLTLFVILGFTAFGFWRRRVEQN
ALISEDAWRNDLQTQDVPGLEYFEMNTIQTATNNFSLSNKLGHGGFGSVYKGKLQDGREIAVKRLSSSSEQGKQEF
MNEIVLISKLQHRNLVRVLGCCVEGTEKLLIYEFMKNKSLDTFVFDSKKRLEIDWPKRFDIIQGIARGLLYLHRDSRLR
IIHRDLKVSNILLDEKMNPKISDFGLARMFHGTEYQDKTRRVVGTLGYMSPEYAWAGVFSEKSDIYSFGVLLLEIISGE
KISRFSYGEEGKTLLAYAWECWCGARGVNLLDQALGDSCHPYEVGRCVQIGLLCVQYQPADRPNTLELLSMLTTTS
DLPLPKQPTFVVHTRDGKSPSNDSMITVNEMTESVIHGR*
>AT1G61440.3
MGKKRIVLLLFISFSYAEITKESPLSIGQTLSSSNGVYELGFFSFNNSQNQYVGIWFKGIIPRVVVWVANREKPVTDSAA
NLVISSSGSLLLINGKHDVVWSTGEISASKGSHAELSDYGNLMVKDNVTGRTLWESFEHLGNTLLPLSTMMYNLVT
GEKRGLSSWKSYTDPSPGDFWVQITPQVPSQGFVMRGSTPYYRTGPWAKTRYTGIPQMDESYTSPFSLHQDVNGSG
YFSYFERDYKLSRIMLTSEGSMKVLRYNGLDWKSSYEGPANSCDIYGVCGPFGFCVISDPPKCKCFKGFVPKSIEEWK
RGNWTSGCARRTELHCQGNSTGKDANVFHTVPNIKPPDFYEYANSVDAEGCYQSCLHNCSCLAFAYIPGIGCLMW
SKDLMDTMQFSAGGEILSIRLAHSELDVHKRKMTIVASTVSLTLFVILGFATFGFWRNRVKHHAHISEDAWRNDLQS

QDVPGLEFFEMNTIQTATSNFSLSNKLGHGGFGSVYKGKLQDGREIAVKRLSSSEQGKQEFMNEIVLISKLQHRNLV
RVLGCCVEGKEKLLIYEFMKNKSLDTFVFGSRKRLELDWPKRFDIIQGIVRGLLYLHRDSRLRVIHRDLKVSNILLDEK
MNPKISDFGLARLFQGSQYQDKTRRVVGTLGYMSPEYAWTGVFSEKSDIYSFGVLLLEIISGEKISRFSYGEEGKALLA
YVWECWCETRGVNLLDQALDDSSHPAEVGRCVQIGLLCVQHQPADRPNTLELLSMLTTTSDLPLPKQPTFAVHTR
NDEPPSNDLMITVNEMTESVILGR*
>AT1G61500.3
MKGAHMMTRFACLHLFTMFLFTLLSGSSSAVITTESPLSMGQTLSSANEVYELGFFSPNNTQDQYVGIWFKDTIPRV
VVWVANREKPVTDSTAYLAISSSGSLLLLNGKHGTVWSSGVTFSSSGCRAELSDSGNLKVIDNVSERALWQSFDHLG
DTLLHTSSLTYNLATAEKRVLTSWKSYTDPSPGDFLGQITPQVPSQGFVMRGSTPYWRSGPWAKTRFTGIPFMDESYT
GPFTLHQDVNGSGYLTYFQRDYKLSRITLTSEGSIKMFRDNGMGWELYYEAPKKLCDFYGACGPFGLCVMSPSPMC
KCFRGFVPKSVEEWKRGNWTGGCVRHTELDCLGNSTGEDADDFHQIANIKPPDFYEFASSVNAEECHQRCVHNCS
CLAFAYIKGIGCLVWNQDLMDAVQFSATGELLSIRLARSELDGNKRKKTIVASIVSLTLFMILGFTAFGVWRCRVEHI
AHISKDAWKNDLKPQDVPGLDFFDMHTIQNATNNFSLSNKLGQGGFGSVYKGKLQDGKEIAVKRLSSSSGQGKEE
FMNEIVLISKLQHRNLVRVLGCCIEEEEKLLIYEFMVNKSLDTFLFDSRKRLEIDWPKRFDIIQGIARGLLYLHHDSRLR
VIHRDLKVSNILLDEKMNPKISDFGLARMYQGTEYQDNTRRVVGTLGYMSPEYAWTGMFSEKSDIYSFGVLMLEIIS
GEKISRFSYGVEGKTLIAYAWESWSEYRGIDLLDQDLADSCHPLEVGRCIQIGLLCVQHQPADRPNTLELLAMLTTTS
DLPSPKQPTFAFHTRDDESLSNDLITVNGMTQSVILGR*
>AT1G61610.1
MAGFNRNLTLVTTLLIFHQLCSNVSCSTSNSFTRNHTIREGDSLISEDESFELGFFTPKNSTLRYVGIWYKNIEPQTVV
WVANREKPLLDHKGALKIADDGNLVIVNGQNETIWSTNVEPESNNTVAVLFKTGDLVLCSDSDRRKWYWESFNNP
TDTFLPGMRVRVNPSLGENRAFIPWKSESDPSPGKYSMGIDPVGALEIVIWEGEKRKWRSGPWNSAIFTGIPDMLRF
TNYIYGFKLSSPPDRDGSVYFTYVASDSSDFLRFWIRPDGVEEQFRWNKDIRNWNLLQWKPSTECEKYNRCGNYSV
CDDSKEFDSGKCSCIDGFEPVHQDQWNNRDFSGGCQRRVPLNCNQSLVAGQEDGFTVLKGIKVPDFGSVVLHNNS
ETCKDVCARDCSCKAYALVVGIGCMIWTRDLIDMEHFERGGNSINIRLAGSKLGGGKENSTLWIIVFSVIGAFLLGLC
IWILWKFKKSLKAFLWKKKDITVSDIIENRDYSSSPIKVLVGDQVDTPDLPIFSFDSVASATGDFAEENKLGQGGFGTV
YKGNFSEGREIAVKRLSGKSKQGLEEFKNEILLIAKLQHRNLVRLLGCCIEDNEKMLLYEYMPNKSLDRFLFDESKQG
SLDWRKRWEVIGGIARGLLYLHRDSRLKIIHRDLKASNILLDTEMNPKISDFGMARIFNYRQDHANTIRVVGTYGYM
APEYAMEGIFSEKSDVYSFGVLILEIVSGRKNVSFRGTDHGSLIGYAWHLWSQGKTKEMIDPIVKDTRDVTEAMRCIH
VGMLCTQDSVIHRPNMGSVLLMLESQTSQLPPPRQPTFHSFLNSGDIELNFDGHDVASVNDVTFTTIVGR*
>AT1G61370.1
MGKIGIVFFASLLFLLIIFPSCAFAAITRASPLSIGQTLSSPNGTYELGFFSPNNSRNQYVGIWFKNITPRVVVWVANRD
KPVTNNAANLTINSNGSLILVEREQNVVWSIGETFSSNELRAELLENGNLVLIDGVSERNLWESFEHLGDTMLLESSV
MYDVPNNKKRVLSSWKNPTDPSPGEFVAELTTQVPPQGFIMRGSRPYWRGGPWARVRFTGIPEMDGSHVSKFDISQ
DVAAGTGSLTYSLERRNSNLSYTTLTSAGSLKIIWNNGSGWVTDLEAPVSSCDVYNTCGPFGLCIRSNPPKCECLKGF
VPKSDEEWNKRNWTGGCMRRTNLSCDVNSSATAQANNGDIFDIVANVKPPDFYEYLSLINEEDCQQRCLGNCSCT
AFSYIEQIGCLVWNRELVDVMQFVAGGETLSIRLASSELAGSNRVKIIVASIVSISVFMILVFASYWYWRYKAKQNDSN
PIPLETSQDAWREQLKPQDVNFFDMQTILTITNNFSMENKLGQGGFGPVYKGNLQDGKEIAIKRLSSTSGQGLEEFM
NEIILISKLQHRNLVRLLGCCIEGEEKLLIYEFMANKSLNTFIFDSTKKLELDWPKRFEIIQGIACGLLYLHRDSCLRVV
HRDMKVSNILLDEEMNPKISDFGLARMFQGTQHQANTRRVVGTLGYMSPEYAWTGMFSEKSDIYAFGVLLLEIITG
KRISSFTIGEEGKTLLEFAWDSWCESGGSDLLDQDISSSGSESEVARCVQIGLLCIQQQAGDRPNIAQVMSMLTTTMD
LPKPKQPVFAMQVQESDSESKTMYSVNNITQTAIVGR*
>Nbe.v1.s00020g25560
MRNINSKKHHFYKYCFFSLLISQILLPILAIPTDTITITNPLTINQTLVSKENKFKLGFFSPGGPNSDKWYIGIWYNEIQD
STIVWVGNRNNPVANSSSHVLKISQNGNLVLVDGVGNSVWSANQESETTAKNTVIAQLLDSGNLVVRHENDENEE
NYLWQSFDYPTDTLLPGMKLGWDLKSGLNRNITSWKSPFDPAPGNYTFKLDINGLPEAFLTNKDDIFYRSGPWNGV
GFSGVPEMKPTEIMAFEFQMNKDQVYYTFQVLDKKICSRLLVKHNGFLERYTWISTSNIWNRFWYAPKDQCDFYEE
CGVSGICNANLSPVCKCLVGYKPKNQVAWDLRDGSDGCVRYHDLDCETDVFNTLKNMKLPETSSSFVDTKMNLD
ECEQKCRYNCSCTAYTTANITGAGSGCVIWTTELVDMRQYSAAEGGQVLYVRVSSSDAAQSGSVGSGDGSGKTKRI
AMATGITAGVVLLLIGVVTIRLLSKRRKLQGPILRKKTEQGGSNQRSQDLLVNTSIIPSKREFSSETSADECELPLFDLS
ALAVATEDFSDANKLGQGGFGCVYKGIIDEGQEIAVKRLSKNSGQGVEEFKNELRLIARLQHRNLVRLLGCCVEME
EKMLIYEYMENKSLDSILFNKQKSVLLDWQRRFNIICGIARGLLYLHQDSRFRIIHRDLKASNILLDKEMNPKISDFG
MARIFGGDETEGNTKRVVGTYGYMSPEYAMDGLFSVKSDVFSFGVLVLEIVTGKKNRGFYYQNNQRNLLGQAWRL
WREETASELLDTAVGESFSSCEVMRCIQVGLLCVQEQAEDRPNMATVVLMLGSESATLPQPKHPGFCLGRRPVDEH
SETIYEETCTVNQVTVTMVDAR*
>Nbe.v1.s00020g25570
MFQKKEFLIILILFIIVLVPFCTSIESISFNQSLKDGDLLISSNKSFALGFFSPGNSNKTYVGIWYNNIPEQTVVWVANRD
NPINSTFGILSINPTGNLEILDKKTKNLVWKTNISSANITEKSYSAQLLDTGNFVLFQDLKKEVIVWQSFDYPTNTILP
NMNFGNDKKTGLNRSLISWKSMEDPGSGEYVYKIEINGTIPQVFLYKNSNRIWRTGPWTGLGWSGVPEMKPGYIFTI

NYVDNDSEASVTFSMLDSAISRLVLNESGMMNILNWQESTQKWVQFWYAPKDPCDNYVHCGKNSNCNLYNLAQ
FECSCLPGYEPSDNRSWYLRDGSHGCLRKKDENICKNGEGFAKVTNVKIPDTYTAILNKSMGLQECEKLCLNNCSC
TAYASANVSVGGIGCITWYGDLIDTREFTDGGQDFYIRVSASTLAQFLKNSNGYHRKRTISIVTVCISAILIAVSIACCLI
IRKKRKDKEDQFTSLITLKRNLASYESSSRGNEIDGSEHVDVLIFDLNTISSASDDFSDANKLGEGGFGSVYKGQLTNG
QEIAVKRLSKTSGQGVEEFKNEVTLIAKVQHRNLVRLLGCCIQKGEKMLVYEYLPNKGLDNFIFDKTKGSQLDWRK
RFEIIVGIARGLSYLHHDSRVRIIHRDLKASNVLLDASMLPKISDFGTARIFGGDQIEANTNRVVGTYGYMAPEYAME
GLFSVKSDVFSFGVLLLEIITGRKNTTHYQDQKLNLVGNVWDFWNDDKAIEVVDPLLGDWYEASEVLRCIQIGLLC
VQSYANDRPIMSEVVFMLCNETKLSNPGQPGFVFRSRNSSSIPYSSSASVGTSVNDISITAQHSR*
>Nbe.v1.s00030g31250
MANGLINFHWFLVLFIVCYFSLLCSASNKVRQGEILRDGNNIISPKGKFNLGFFSPKHSNQRYLGIWYADVPVLSVV
WVANREKPVCDQNGVFTIEKNGNLVVKDGRGDLLWSTNVSVVETINSTVSLLDTGNLVILNDNNKVLWQSFQHST
DTFLPEMRVYMDGVLRSWTSESDPSPGRYSLGVDPRASPQIVIWDGSNRHWRSGYWDGLSFTGVPDMKALYFNGF
KLYNEGNRLYFTYTAANTSNLVRFHISPSGYEQQQMWDMDKRKWSMIQSHPLGYCDIYNSCGNFAKCDISNSRKC
TCLYGFVPKDWEQWNARNWSGGCVRRTRLECGRNSSVLRNDIGNGDGFLEIKGIKLPDFADTATAENVDECKSTC
LENCSCTAYAFVIGIYCMIWSGDLVDLQQFNEGRNTLYVRLAHSEFGKKNRTIKIVLISILVAVAFVICMAIWLLCKYK
AKRRGSIRINEMPIRDPIRSGELPMDLSGPGDLSVEGHQGSSSELKFFSFSSIVAATRNFSNENKLGQGGFGPVYKGKL
QCGEEIAVKRLSRKSGQGVEEFKNEIMLIAKLQHRNLVRLLGCCIEGEEKILLYEYMPNKSLDSFLYDPAKQAQLNW
RTRFNIIEGIARGLLYLHRDSRLRIIHRDLKASNILLDEEMNPKISDFGMARIFGGNQNEANTNRVVGTYGYMAPEYA
MEGSFSGKSDVYSFGVLLLEIICGWRNTSFRSDQHSSIIGYAWEKWDEGRPTDLVDRSIWDECQHNEVLRCIHLALLC
VQDMAVHRPNMSSVVLMLETDNIRLPLPRQPTYTSMRKHENADIWNEKQEFSSNDVTMSVIVGR*
>Nbe.v1.s00040g37970
MLKYTPLFSSHRMKATSNEGFKLFFLLCLFSIYQILGATDTITTTQFLKDGELNISSSGGNFEMGFFSPGNSRFRYFGIW
YKNISVTTVVWVANREAPLSNTSGTVKVIKPGVIVIVNETNHIIWSTNSSRSVQNPIAKLLNSGNLVVKEADVDDDK
AGSFIWQSFDYPTDTLLPSMKIGFNFVTGKEVYLSSWKNEEDPAPGDYTYHCDPSGYPQNILKKGSNVIYRSGPWNG
LRFSGATNSRESQFYTFGVFSTKTEVYFSYHLLASVVTRFILNQNGVLQRWTWGDRNKGWALYLALPTDNCDTYRL
CGGYGSCNSLNSPVCGCLDKFVPRIPEDWKKADWSSGCVRSAELNCLKGDVFLKYSKLKLPDTRNSWFNASMNLE
ECKKICLKNCSCMAYSNLDIRNGGSGCLLWFEDLLDIRQLANEGQDIYIRMAASELTSQDKSNGHKGKLLAFIIPLSA
GVVLIFLSLVIFLRRRKRASEKKKGFWGCNYKMDYHTGNQSEEFELPLFDLSTIAKSTYNFSEESKIGEGGYGPVYKG
VLEQGQEIAVKRLSKTSTQGQDEFKNEVMYIVKLQHRNLVKILGCCIEGEEKMLIYEYMPNGSLDSFIFDDAGSRVLD
WPKRFHIINGIARGLMYLHQDSRLRIIHRDLKANNILLDNDMNPKISDFGIARSCEDDKFGAKTHRVVGTYGYLSPE
YAVHGVYSVKSDVFSYGVLVLEIVSGKGNRRFSHPDHNLNLLGHAWKLFKEGRSMELLGFPIDVHSTPEVIRSIHVG
LLCVQHCPEDRPSMSSVVMMLNNEGELPLPKQPGFYVEANAPDNEFSSSQYAHSTGNEITITVLDAR*
>Nbe.v1.s00060g33290
MAASFRVSVWFVFFFLLSCLSLFCSANDRVTVGEILKDGDNITSKEGNFVLGFFSPTGSSKRYLGIWYADVTVQTVVW
VANRHKPVLDKNGTFLIDQIGNLVVKNGHGDSLWSSNVTVATKNSTACLLNNGNLVILNNDRSAARLNTELWQSF
LHPTDTFLPGMRVFVERQSKEHKVFSSWTNESDPSPGRYSLGVDPRGAPQIVIWDGSDRRWRSGHFDGVEFTGVPN
VTRSTLFSGFKINNEDGKSFFTYTPPETSLFVRFQITVTGNELQQRWEEDKEQWSTLQSMPMGGCDLYNFCGNFAEC
HEQVCLCLEGFVPRVEEQWHAGNRTGGCIRRTELECRKSSSVPRNDSAKDDGFLAVRRVKLPDYADIIADAQNTDA
CKTKCLSSCFCNAYAFVKGINCMIWTEDLVDIEQFEEGGNTLYVRLDPSELGKNNRTIIIIVVSVLVALALVVMAAVW
LICRYRARKQESKRINEIPKNHLVRSGEFSADLSGPGDLNAEGHQGNGSELAFFSFSMVATATDGFCLANKLGQGGF
GPVYKGRLPCGQEVAVKRLSQRSGQGDEEFKNEIALIAKLQHRNLVRLLGCCIEGEEKILLYECMPNKSLDTFLFDPV
RKSQLDWRKRFSIIEGIARGLLYLHRDSRLRIIHRDLKASNILLDEEMNPKISDFGMARIFGGNQNEANTNRVVGTYG
YMAPEYAMEGLFSGKSDVYSFGILLLEIISGRRNTSFRSDEHSGIIGYAWEKWDEGRPMDLVDRSIWDGCQHDEALR
CIHLAMLCVQDLAVHRANMSSVVLMLETDNMRLPLPRQPTYTSMRRSVDADMWHGNQDLPSSNNVTISVLIGR*
>Nbe.v1.s00090g06980
MGFCQAWKILLISLLLYCIIQSYYCYASDTILQSQKLLVGNTLTSASQVFELGFFSPANSSKRYMGIWFKNIPSHKVVW
VANRENPLNVSDSAASLSISKNGNLVILDGVLNVIWSSNVFVPTNNTVEVVLQDSGNLVLKDNISGQCFFESFDYPC
DTFLPGMKIGFNRITGERWLLSSWHKENDPSPGNFSIGISDQLPPQFFIWNKSTPYYRTGEWNGLKFTGLPYIDSAAYI
VQFVFQQDFQEGTAYFTFLPNTSFLTFVELQSTGSVQVVQWTDGAPAWEVYAKMVHPPCDIYNTCGPSAICSKNKY
PTCSCLRGFVPRSSDEWSRGNWTGGCVRRTKLLCQRKGINTSPGVGQKDGFLKFSGLKLPDLAAIFRLDSASECERL
CLNNCSCTAYAYVAGIRCMLWSGDLLDMQDYSYSGEDLFLRLAYSELGQKRKGTISFRIGDSFNISKEYALESFWVGN
LKKEDPIELPLIEFELIAAATNNFKVDNKLGEGGFGPVFKGKLKGGQEIAVKRLSNRTGQGIEEFKNEIVLISKLQHRN
LVRLLGCCIEGEELIIYEYMPNRSLDKFLFDTSQKELLDWPKRFNIIQGVARGLLYLHRDSCLNVIHRDLKVSNILLDE
EMNPKISDFGLARTFQKQQQLVHTHRVAGTYGYMSPEYALRGVFSEKSDVFSFGVLLLEIISGKKNSSFHYVEENLNL
LNYAWKLWSEQRGLDFMDGTLINSFSPEEITRCLRVGLLCVQECPRDRPTMAAIILMLNSEMKCSTPKQPTFKFETYL
DLGSSAKDNDKCSVNEFSASLSQGR*
>Nbe.v1.s00110g41560
MPSLFVYLFVLSYISLKTPLPFVTSQKNSVIGDTIDANKWISIASTIVSSGGNFEMGFFTPGNNSNYYIGIWYKKLSIQT

AIWVANRVTPVSIYEMDFAQFKIQNGNLVLFNGTRHIIWSTSLNFSTNSNTSQVVATLFDDGNLILSNGDINNSRNHI
WTSFDHPSHTFMPGSKFGYNKRTKLKQALISWKNANNPSPGPFTHEVNMDKYSGEGVNMWNDSEIYWKSGPWT
GNNFTGVPYEQNPMFNYTYVNNDNEVYYQYNFFNPALISRFIMDFDGQTKQFLWMDSTNEWNVFYADPKQVCD
VFGYCGAFGICNELSSAASVCDCLPGFKPKSDEDWNLKSFSGGCMRKIGLKCSNFSVVDGEKDKFLMQKNMRLPA
NNESMRVQNEVECENTCLKNCDCVAYAYNNGCLIWRGEIFNLQQLSQDNANGSTIYVRLAASEFSSNKDEHQKQT
SKKLKIAIPIGVIAALLFLSCFSYIYYRKRRSTKIKESTTKSLMQNTEGEAELIDIQDDDKGNIEVPFFSFESILMATDDFS
EQNKLGQGGFGPVYKGIFSGGREIAVKRLSSLSGQGINEFKNEVMLIARLQHRNLVRLLGYCVQSNEKILLYEYMAN
KSLDTFVFDRKLCMKLDWKKRFEIIEGIARGLLYLHYDSRLRIIHRDLKTSNILLDEELNPKISDFGLARVVEGKITQA
NTNKVVGTYGYMAPEYAIDGLFSIKSDVFSFGIVILEIISGRRNTGFFHQEETSNLLGHAWRLWKEDKAMDLVDQSL
QELCNKEEAIKCLNIGLLCVQEDPKDRPNTTNIIMMLGSENIISLPRPNQPAFMTRKCANNTSSSSAAKSDVVSNNE
LTVTIEIGR*
>Nbe.v1.s00190g10340
MKRFAGFYFLSCNIVSLLFIISTALDTISKDSPIEDGHTVVSADGYFELGFFSPGNSTNRYVGIWYKKISKAQKVVWVA
NRSNPLNDTSGVLRVSDKGILLLMNGTQDVIWSSNSSTSLRNPVAQLLDTGNLVLKDGTDLVWQSFDYPGSTLLPG
MKVGRNLVTGLNWTLTSWRSNDDPSPGEYMHRVDTNGYPQYFLLEGPVIKYNTGPWNGRSFTGGPNLKRNPYYT
FEFVMNDKEIYFTYELLNSSLPTWVVLNPAGLIQRLLWIERTESWFLYSTGQMDNCDRYALCGQFGKCNINDSPPCD
CLRGFKPKYQQEWDAADWSNGCVRRTPLACGTSDRFLKYSGVKFPDTRHSWFDKTIGLEECQRLCLRNCSCVAYS
NLDVRNGGSGCLLWFNQLIDIREYAQLDEDLYVRMAASEIGSNHIGNTIKAVIAIVSTVSAIIVLGFLSWSVMRRKKR
KRAGQEGKEEMELPLFNLMSINIATDNFSSDNILGEGGFGPVYRGKLSAGPEIAVKKLSQHSGQGLEELKNEVVLISK
LQHRNLVKLLGCCLEGEERILVYEYMPNNSLDFFIFDESRKRQLPWENRFQISMGISRGLLYLHQDSRLRIIHRDLKTS
NILLDSELNPKISDFGLARIFGGDQIEEKTKRVIGTYGYMSPEYAVDGKFSVKSDVFSLGVLLLEIVSGKKNRTFQHSD
HHHSLLGHAWLLWNEGKALELMDGCLKESFVESQVLRCIHVGLLCVQKLTVDRPTTASVVFMLSHEEVPLPQPKE
PGFFIERNASETDDSNEKRFLTDNVLTLSILDPR*
>SolyMTch02g054880.1
MKLSTREILLFPVVLVLFLSISTASGIISTNKFLRDSETLVSNDKRFIFGFFSLENSTNRYVGVMFNVQPPTVVWVANRE
RPLQDSRGRVTISDDGNLVILNSQNRSIWSSNISPAVRNSTAQLLDTGNLILNDSSNGRVLWESFRDPSDCFLQTMKIG
VDVSTNTTNLLKSWISPSDPSVGSFSAGIQPETVPQISIWKNGKPHWRSGPWNKQVFIGVPDMTSFYLNGFDLVNDN
KGTVYLTYLYANQVELMYFTLNSTGFLQQKYMDPSKNDWEVTWEFPATECDFYGKCGPFGSCDPTSSPICSCLEGFK
PTNEEEWRKGNWTRGCNRKSMLESERNSSNLEQGKQDWFLKLQSMKVPDSAIWVPFVDEDCVNGCLRNTSCIAY
SYYTGIGCMHWEGNLLDVQKFSMGGVDLFLRLSYSERDQKREYKVIIAIIVPVGSIILAIFGYISCKYVAKRRGWKRM
SKIFLSESSPNYYKEDKITEDINQAKLEELLVYNFDILANATENFHLSSKLGQGGFGPVYKGKLPDGQEIAVKRLSQSS
GQGLQEFMNEVVVISKLQHRNLVRLFGCCIERGEKMLVYEYMPKRSLDAYLFGSQQQAEEFLDWSKRVIIIEGIGRG
LLYLHRDSRLRIIHRDLKASNILLDEYLNPKISDFGMAKIFAGNQDQANTSRVVGTYGYMAPEYAMEGRFSEKSDVY
SFGVLLLEIISGRRNTSFHQDDGALSLLAWAWKCWIGNKIVELVDPKITELHLGKEIVRCVQVGLLCVQEYAEDRPN
VSTILSMLTSEIDNLPSPKQPAFTTRPSFSKKGTSKSQGSVNNVTVTIMEGR
>SolyMTch02g054890.4
MKLSTREILLFPVVLVLFLSISTASGIISTNKFLRDSETLVSNDKRFIFGFFSLENSTNRYVGVMFNVQPPTVVWVANRE
RPLQDSRGRVTISDDGNLVILNSQNRSIWSSNISPAVRNSTAQLLDTGNLILNDSSNGRVLWESFRDPSDCFLQTMKIG
VDVSTNTTNLLKSWISPSDPSVGSFSAGIQPETVPQISIWKNGKPHWRSGPWNKQVFIGVPDMTSFYLNGFDLVNDN
KGTVYLTYLYANQVELMYFTLNSTGFLQQKYMDPSKNDWEVTWEFPATECDFYGKCGPFGSCDPTSSPICSCLEGFK
PTNEEEWRKGNWTRGCNRKSMLESERNSSNLEQGKQDWFLKLQSMKVPDSAIWVPFVDEDCVNGCLRNTSCIAY
SYYTGIGCMHWEGNLLDVQKFSMGGVDLFLRLSYSERDQKREYKVIIAIIVPVGSIILAIFGYISCKYVAKRRGWKRM
SKIFLSESSPNYYKEDKITEDINQAKLEELLVYNFDILANATENFHLSSKLGQGGFGPVYKGKLPDGQEIAVKRLSQSS
GQGLQEFMNEVVVISKLQHRNLVRLFGCCIERGEKMLVYEYMPKRSLDAYLFGVHIEEEYFLDWSKRVIIIEGIGRGL
LYLHRDSRLRIIHRDLKASNILLDEYLNPKISDFGMARIIAGNQDQANTIRVVGTYGYMAPEYAMTGRFSEKSDVYSF
GVLLLEIISGRRNTSFYQEDGALSLLAWAWKLWNENKIVELVDSKIIELQLKKEIHRCVHVGLLCVQEYAEDRPNVST
VLSMLTREIDDLPSPKQPAFTTRPTPSKKGSSRIQVSVNDVSITIMEAR
>SolyMTch02g054920.2
MGFRQAWKVLLICLISCYIIQTYYCNASDTIQQSWKLLVGETLTSASQVFEFGFFTPANSDKRYLGIWFKNIPPIKVVW
IANRESPLKVSDSAASLSISENGNLVLLDGTQTVIWSSNVSISSNKTVAVVLLDSGNLVLKDNVSGQTFWESFDYPCD
TFLPGMKIGFNSKTGEKWLLSSWQKENDPSPGNFSIGISEQLSPQFFVWNKFTPYYRTGEWNGLKFIGLPCIDSAAYII
QFVFQQDFQEGTTYFTFLPNTSFLNFVELQSTGSVQVVQWTNGDPAWEIYATMVRAPCDIYNTCGPSAVCSKHNFPI
CSCLRGFVPQSGDEWSKGNWTGGCVRRTELLCQQKGNTLSPGVGLQDRFLKLSGLKLPDLAAIFRLDSASECEKLC
LNNCSCTAYAYVAGIRCMVWSGDLLDMQDYSYSGEDLFLRLAYSELVFPGKRKFKRTLIICSAVFSCLFSGFALFCLLK
HKIHQTGQKRKGARSFSLGDSCYISKDYTVESLWIGNLKKEDPIELPLIEFEVIASATNNFNEENKLGEGGFGPVFKG
KLKDGPEIAVKRLSNRTGQGIEEFKNEIVLISKLQHRNLVRLLGCCIEGEEFLIIYEYMPNRSLDKSLFDASQKELLDW
PKRFNIIQGVARGLLYLHRDSCLNIIHRDLKVSNILLDEDMNPKISDFGLARTFQKQQQLVHTHRVAGTYGYMSPEY
ALRGVFSEKSDVFSFGVLLLEIISGKKNSSFHYVEENLNLLNYAWKLWSEQRGLDFMDGTLINSFSPEEIPRCLHVGLL

CVQEHPRDRPTMADIILMLNSEMKCSSPKQPTFKFETYLDLDGSAKDNERCSVNEFSASLSQGR
>SolyMTch03g080750.1
MKREWHCQDGIFFLFLMLRIMHLPLVYAENDTLTQSQQLSLNQTLVSAGNIFELGFFSPSNPSRLYLGIWFKGIPGQRI
VWVANRENPLTAAILKIGGDGNLRIMDSNIQNIVWSTNVAVESSCTVAVLTDEGRFILKDNVSGSSLWDSFNYPCDT
LLSGMVIGYNTRTGVKLALSSWQAEDDPSPGKFIAGLSVDMPPQGFIWTSYSRPYWRGGPWDGGSFIGIRDPDKGY
ASGINIVSDKHQGTAILSFNTFINSHVTIVVLKPSGLLQIMYWEEESNVWKVTWEGPDNPCDVYGACGPYSVCDKN
KSPVCDCLRGFVPKSTDEWIRGNWTGGCVRRTKLLCEISTSDIAPKESKNDKFLKLREMKLPDYYTYLYDQNGIQNC
EKWCLNNCSCAAYAYPDGINCMVWTSELIDVQQFPYNGANLFLRLAYSELDEDNGKAKLIIGLATVSSILLLSILGCI
FCKWKANKRGSEATDYLWEEQALLKDSSELHLLDFSKLAVATDNFNEINKIGAGGFGPVYKGKLEDGQVIAVKRLS
SFSGQGIEEFKNEVLLISKLQHRNLVRILAYCVHGKEKLLVYEYMANRSLDTLLFDPKRSHRLPWPKRLDMIYGIARG
LLYLHRDSCLRVIHRDLKASNILLDGDMNPKISDFGLARTFQVTQELANTHRIVGTFGYMSPEYAMGGLFSEKSDVY
SFGVLLLEIVSGRKNNNFYDNDRHFNLLSYAWKLWTESKGLDLMDKSISDSRSAATVLKCIHIGLLCVQDHAVDRPL
MSSVVLMLRSKMDLPQPKQPKFIFKRWLNSDAQSQSSKARSINGITISSAEGR
>SolyMTch03g080760.4
MIRKGKKMVWPRWDFFLFLMLGRMLLAQLSAASDTLTQSQQLSLNQTLVSAGNIFELGFFSPRSSRSLYIGIWFKNIS
RRRVVWVANREDPLQASDSDTILKIGGDGNLIIMDGNQNIIWSTNISIQSNKTSAVLTDKGEFILKDDVTGSSLWDSF
NYPCDTLLSGMNIGYNTSAGVRLVLSSWQAENDPLPGKFTSGLSVEMPLQGFTWTNYSRPYWRGGPWDGANFIGI
PDVDKGYASSINVIVNKQQESGFLSLNNFNDSDVIIMVLKPSGLLQTILWVEELNAWQVTWEAPGNPCDVYGTCGP
NSVCDKNKSPVCDCLKGFVPKSTDEWIRGNWTGGCVRRTKLLCEISTSENTTNGYGSDNFLQLREMKLPDHYTYFY
AYDYQSCKEWCLNNCSCAAYAYPDRIDCMVWTSELMDVQQFPSDGVDLFLRLAYSELDEDKRKKKLIIGLTTLSSILI
LGILGYIFCRWKVNQRGNRRNRVEHHIPADKCQISSEMSTDNLWEEQELPKDSSELPLLDFAKLATATDNFSEINKIG
AGGFGPVYKGKLEDRQMIAVKRLSSQSGQGIEEFKNEVLLISKLQHRNLVRILAYCVHGKEKLLVYEYMANKSLDTL
LFDSKKSHQLPWPKRFDMIQGIARGLLYLHRDSCLRVIHRDLKASNILLDDDMNPKISDFGLARIFQVTQELANTNR
IAGTFGYMSPEYAMGGLFSEKSDVYSFGVLLLEIVSGKRNSGYYDHERHHNLLSYAWQLWTESNGLDLMDKSILDS
DSSATVLRCIHIGLLCVQDHATDRPSMPSIVLMLSSEMDLPQPKQPTFIFQRWLNSDTQSQISKTQSVNDITVSVAEGR
>SolyMTch04g130950.4
MTTTSRNVQHFVHVILVFLHCFNTGFCTEIDSITSTLSLRDPGILSSPGGVLKLGFFSPLNSSNRYVGIWYNFSETIVIWV
ANRDKPLRDSSGVVKISGDGNVVVMNGEEEILWSSNVSTSQVNSIALLQDSGNFVLVDHLNNGSTIWQSFEHPSDSI
VPKMSISENTRTGERVEVKSWRSPWDPNFGNFSLGMNSGFIPQVYIWKGSQPYWRSGQWNGQIFIGVQDMYSVSSD
GFNVVNNREGTVYLTGPGDFDFLTKFVLDWKGNLVQSYWDANETTWKIIWSAPNNDCEVYGMCGPFGSCNHLES
PICSCLKGFEPKHREEWEKGNWVSGCLRRKALQCEVRNNSGDSSKEDGFLKIGSIKLPDFSERSSTREDQCRSQCLG
NCSCIAYAYDSGIGCMSWNNNLIDIQQFQSRGEDLYIRMAHSELDHHKDIKKIVIPVILGFLTLCVCLFLCCTRMARR
RGVKRKKINLLGDRSAVHMEELPVFSLDTLANATSQFHEDKKLGQGGFGPVYMGKLEDGKEIAVKKLSKASGQGL
EEFMNEVLVISKVQHRNLVRLLGCCVDKEEKMLIYEYMPKKSLDVFLFDEGHRGILDWRKCSTIIEGVGRGLLYLHR
DSRLKIIHRDLKPSNILLDNDFNPKISDFGMARIFGSDQDQADTRRVVGTYGYMAPEYAMKGRFSEKSDVFSFGVLV
LEIISGRKSTSSWNETSSFSLFGYAWMLWKEQDLSTFIDPFILNPSSEMEIKKCIQIGLLCVQEFAEDRPSISSVLAMLTSE
TTSIPTPSQPAFTERHDCIFKMCNETNCTLNNISITNITGR
>SolyMTch04g124180.1
MSNSSSRRNLQYFVHIILVILRFVDTGLCSEVDNITSIQSLRDPGILSSPGGVFKLGFFSPQNSSNRYVGIWYNFSVTTVI
WVANRDKPLRDSSGVVKISRDGNIVITNGEEEILWSSNVSTSQVIIPIGLLQDSGNFVLVDHRDMSTIWQSFEHPSDST
IPRMRISENTRTGEMVEATSWRSPSDPNIGDFSLRMNSGVIPQVYIWKGRRPYWRTGQWNGQIFIGVQNMYSVVSD
GFNVVDDREGTVYFTGPTRDNFLRILVLDWRGNLVQSYWDVNETKWKIIWSAPNNDCEVYGTCGPFGSCNHLESP
VCSCLKGFEPKHMEEWEKGNWTSGCVRRSALQCEVKNNTTDSSKEDGFLKMELMKLPDFAERSSTTEDVCRSRCL
GNCSCIGYAFDSSIGCMSWSIMIDIQQFQSSGKDLYIHVAHSELVFSADHRKEYIKKIVIPVIVGSLTLCVCLFLCYTMM
VRRRGVKREEVLLGNKSPVNMEELPVFSLDTLVNATSQFNEDNKLGQGGFGPVYKGILEDGKEIAVKRLSKASKQG
LEEFMNEVLVISKVQHRNLVRLCGCCVDEEEKMLIYEYMPKKSLDVFLFDEGHRDILDWTKRSIIIEGVGRGLLYLHR
DSRLKIIHRDLKPSNILLDNNFNPKISDFGMARIFGSDQDQADTMRVVGTYGYMAPEYAMEGRFSEKSDVFSFGVLV
LEIISGRKSTSSWTETSSLSLMGYAWKLWKEQDLSTFIDPFILNTSSEMEIRKCIQIGLLCVQEFAEDRPNISSVLVMLTSE
TTSLPAPSQPAFTERRHFRMCNENRETKFTLNKMSITNLTGR
>SolyMTch07g222000.2
MKGLFSLCICYQFLFILLTSAALDTITTDKSIRDGDTIVSAGGVYELGFFSPGNSKNHYVGIWYKKISNGTVVWVANR
SIPLNDTSGVLTLNPNGILVLVDKSNVSIWSSNSSRLLKNPKARLLDSGNLVVSDGNDRGLENNFAWQSFDYPGNTL
LPGMRLGKDFVTGMNWHLTSWKSTDDPTPGDYVDRVDSHGYPQLFVWKNSSIVFSSGPWNGIAFSGSPNNKPNT
YYSFEFVINQQEIYYTYTIKNDSIPTRVVLNPSGVLEHLTWIERSQSWFLYLTAQFDNCDRFGLCGPYSSCNINNSPPC
DCLKGFEPRYPQDSATEWSSGCIRRTSLDCTHDGFLKFSGIKMPDSRNSWYNDSMNLEDCEKMCLADCNCTAYSDL
DVRNGGSGCLLWFGELIDIRGFSQNEQNLFVRVAASELDRKGRRKRAALIGVISAVVATFILSFLAWFYFRRRKRRRG
LEVENEDMELPLFDLVTVTTATDNFSSANVIGEGGFGPVYKGILPNGQDIAVKRLSKHSGQGFQELKNEIVLISKLQH
RNLVKLLGCCLEGEERMLIYEFMPNASLDYFIFDSSRKASLAWKNRFEIAMGISRGLLYLHQDSRLRIIHRDLKTSNIL

LDTDMNAKISDFGLAKIFGGDQVEGKTKRVIGTYGYMSPEYAVDGKYSVKSDVFSIGVIILEIVSGRKNRKFRHLEHH
HNLLGHAWLLWIEGNALELIDECIKESFSESQVLRCIQVGLLCVQKLPEDRPTMASVVFWLGNEGLVLPQPKQPGFF
IERNSMESTKSSTDEGYLSNNVSITILEPR

Supplementary Text S3. Gene sequences of *StRDA2-D*.

>A157_02G016870.2_(*StRDA2A*)

ATGAGCATATCTATGCTTTTTCATCTTTTACTCTATTGTTTTTATGTAGTTTTTTCCAGTGCCAATACTTCAGACAC
CATTAGCAGTAGTGAGCCTGTGAGAGACTCAGAAACTGTATTTTCTAGAGGCAAAAGATTTAAACTGGGTTTT
TTTAGCCCCAGGAATTCTGCAAAACGTTATGTAGGGATTATGTTTAACCTACCATCACCAACACCAACTGTAGT
ATGGGTAGCTAACAGAGATGAGCCTTTACACGATTCTAGTGGAATACTCACAATATCAGAAGATAGCAATCTT
GTCATGTTGAATGGACAGAAGGAGATAATTTGGTCATCCAGTATTTCAAACTCTATGAAGAATTCCACAGCAC
AACTCTTGGACACTGGAAACTTAGTCTTGAAAGACAGCTCAAATGGGAGAGTTCTATGGGAAAGCTTTCAAT
ATCCTACGGATTCTCTCTTACAGCTCATGAAAATGGGCACTGATAAGCGTACTAACACAACAACTCTCCTGAA
CTCATGGGGGAGTCCTGATGATCCATCCGTTGGGAGCTTTTCAGCTGGAATTCAACATCGATACATTCCTCAGT
TTCTTATTTGGAAAAACAGTTCTCCTTACTGGCGGGGCGGTCCATGGAATAAACAGATATACATAGGATTACC
AGAAATGAATTCTTTCTATCTCTATGGTATTGACCTAGTAATTGATAATGCTGGCACAGCATACCAAACTTATTC
AGACCCAAATCAGTCTAGGATACTCTATTATTCCTTGAATTCAACAGGGTCTTACCAGGAGAAGGTTTGGGAT
CAAAGTAAGAAGGATTGGGTGGTAACATGGGCAAATCCCCGAACAGAGTGTGATTTTTATGCCAAGTGTGGG
ACATATGGAAGCTGTAATCCAATGAATTCTCCAATATGCAGTTGTATACAAGGTTTCAAACCAAAAAATGATG
GAGAATGGAAGAAGGGAGATTGGTCTGGTGGATGCATCAGAAGTACTGCACTAGACTGTGAAAGGAACAAA
ACTGATATTGAGAAGGACAAGCAGGACGGGTTTCTGAAGCTGCAGACAATGGGAGTGCCAGATTTTGTAATT
TCGGTACCTTCTGCAAAAAAAGACTGTGAGAGTGACTGTTTGAGTAACTGTTCCTGCACTGCATATTCATACT
ACAGAGGCATAGGTTGTATGCATTGGAATAGAAGCTTAGTTGATATTCAAGAATACTCCAGGGATGGGGCGGC
TAGTTTGTTCATTCGCCTTGCCTACTCTGAACTTGGTAACATACTTCTTTACAACCTTGAGCTTTCAATTGTTTAG
CTTGTTTTTTTTTTTTTGGAACTACAAGGAGTTGAAATTTAAAGTCTTCTTGTCTTTGAAGAATATAACCAAGAC
TCCCAATAAAATCTATAAAACCATCGAATTAGGCTCAAATTCTCAATTAATGTATTGCTTAGCTCTTTTCTATTTT
TGGTGTAAACAAAGATATATTAACTAGGACAGAAGACATCTTTAATGTGCTAGATTGTTTATAGGCAGCAGTTT
AGATTAAGACTGTTTTATTCTGTAAGTTGTACTCCCTCTGTCCCAATTTATGTGACTTACTTTCCTTTTTGGTCAG
TCCCAAAAAGAATGACACATTTCTATATTAAGTAATAATTTAACTATAAAATGTTTATTTTACCCTTAATGAAAT
GATTTACAGCCACACAAATTTCTATCATTCATTTTGGACCACAAGTTTTAAAAGTCTTCCTTTCTTTCTTAAACT
TCGTGCCGAGTCAAAGTACCTCACATAAATTGGGACGGAGGGAGTAATACTTTTGAAGGGGAACTGCACATT
TTTTGTAGTGCACCTGTGTATATATAGACTAAAGTTACCAGTTTCAAAAAAAATAAAAATCAATTAGTTCTATC
ACTTCCATGGAAAGAAAGATATGCTCAAGAACAGTCTATGGCTCATACATGTTTGCAGCTGCAAATGACAAG
AAAGACATTCCTGTAGCAGCTATTGCAACCACAGTCTCAATAGGCTCAATAACAGTTATCTTGTGTGGATATCT
TTTCTGGAAATTGTTGGCTAAGCACAGAGGTAATTAATCAAATAAGATTTTTGAGAAGACAAACTAGAACTTC
TCTGTCTGCAAATGGTTTGATTTCTGAATGTCCACTCTGAACCAGAAAGGAAGAGGAAAAATGAAGCACTCT
TAAGAGAAGCATCTCCAAAATTTTACCAAGAAGGCATGAGTAAAGATGACATCAATCAAGTCAAAATTGAAG
ACATCACCTTGTACAGATTTGACATGTTAGCGAATGCCACTGACAGTTTTCACCCAGCTAGCAAGCTGGGGCA
AGGAGGCTTTGGTCCAGTCTACAAAGTAATGTTTCTTGATGCATCTCTGCAGTGTTCTGACAAATGAACACAA
TATAAAATAAATTAGATCAATTATCTGCAGGGACAATTGCCAGATGGACAAGAAATTGCAGTAAAAAGGCTTT
CACAGTCTTCTGGTCAGGGGCTACAGGAGTTTATGAATGAGGTCGTTGTGATTTCTAGACTACAACATCGTAAT
CTTGTTAGACTCCTTGGCTGTTGCACAGAGAGAGGGGAAAAGATGCTGATTTATGATTTCATGCCAAATAGAA
GCTTAGATGCATATCTTTTTGGTCAGTGCAGAATTTTCTTTCATTGGTTTTGCCTGTCCTTCAACTTAAGTACTTC
CAGAATCAAAAGAAAGCTGTATTTCTTTAAGGAAACAAAATCCATCATACATTTCTCGGTGATACCTTTTCAG
GTTCACACCAGGAAAAGTTCCTTGATTGGAGTAAACGAGCCATCATTATTGAAGGAACTGGTCGAGGCCTCC
TTTACCTTCACAGAGACTCAAGACTACGAATTATTCATAGAGATCTGAAGGCAAGCAATATCCTGTTGGATGA
ATACCTAAACCCAAAAATTTCGGATTTTGGCATGGCGAGGATTTTTGGAGGCAATCAAGATCAGGCCAGCACT
ATAAGAGTTGTTGGCACATAGTATGTTTGCACTGCTTCCTCCATTTTTTCATGGATGAACTGCCATCATTAGTCA

TTGAGTTTCTGTGTAATATCTTATGCAGTGGTTACATGGCCCCTGAATATGCAATGTACGGAAGATTCTCAGAA
AAATCAGATGTCTACAGCTTCGGTGTGTTGATATTGGAAATTGTCAGTGGAAGGAAGAACTCTAGTTTTTATGA
TGATGAAGATGAACTGACTCTACTTGCATATGTAAGTTCATTCTTGCAACTATATATAAAGCTTTAATGTTCTGC
TAGCTCATCAAACCAAAAAAAATAGAGGAGAAAAATTATGAAACTTCTGAAAATAACATTTTTGCTGTTTGAC
AATGTCTTTGTTCATTAGGCATGGAAGTTGTGGAATGAAAACAATATCATAAAATTGATAGACCCCAAAATATT
TGATTCAAGCTTTGAAAAACAGATGGTGAGATGTGGACATATTGGATTATTATGTGTTCAAGAATATGCAGAA
GATAGGCCAAATGTCTCCACAGTTCTGTCAATGCTCACTAGTGATATTGCTGAACTACCTACTCCTAAACAACC
TGCATTTACCGGAGGACATGCTTCACCACAGCAAGTATCTTCTAAAAGCCAAGGGTCCGTGAATGCTGATACC
ATTACTATATTGGAACCACGA

>A157_02G016830.1_(*StRDA2B*)

ATGAGCTTATATCGATCATTTCTTCTTTTGCTTTGTTGCTTTTATGTAGTTTTTTCTGGTGCCAATGCTTCAGACAC
CATTACCAGTAGTGAGCCCGTGAGGGACTCGGAAACTGTCTTTTCCAGTGGCAAAACATTTAAACTGGGATTT
TTCAGTCCTGGGAATTCTGCAAATCGTTATGTAGGGATTATGTTTAACCTACCATCACCAACACCAACTGCAGT
ATGGGTAGCTAACAGAGACAAGCCTATAAATGATTCTAGCGGATTACTCACACTATCAGAAGATGGCAATCTT
GTAATCTTGAATGGACAGAAGGAGATAATATGGTCATCCAATATTTCAAACTCTATGAAGAATTCTACTGCTCA
ACTCTTGGACACTGGCAACTTAGTCTTGAAAGACAGCTCAAACGGGAAAGTTCTATGGGAAAGCTTTCAATA
TCCTACAGATTCTGTCTTACAGCTCATGAAAATGGGCATTGATAAGAGTACTAACACAACGGCTCTCCTGAAA
TCATGGAGAAGTCCTGATGATCCATCTGTTGGGAGCTTCTCAGCTGGAATTCAACTTCAATACATTCCCCAGG
CTTTTATTTGGAATAACACCGTTCCTTACTGGCGTAGTAGTCCATGGGATAAACAGATCTATATTGGATTACCAG
AAATGAAATCTTCCTATCGCTCTGGTGTTGACCTTGTAGCTGATAATGCTGGCACCGCATACCAAACTTATTCC
AATGGAAATCAGTCTTGGATACTCTATTATTCCTTGAATTCAACAGGGTCTTATCAGGAGAAGGTTTGGGATCA
AAGTAAGAAGGATTGGGTGGTAACATGGGCAAATCCCCGAAGTGAGTGTGATTTTTATGCTAAGTGCGGGGC
ATTTAGTAGTTGTAATCCAAAGAGCTCTCCAATATGCAGTTGCATACAAGGTTTTAAGCCTAAAAATGAAGGA
GAATGGGAGAAAGGAGAATGGTCTGGTGGATGCATCAGAAGGACTGCATTAGACTGTGAAAGGAACAAAAC
TGATGTTGAGAAGGGCAAAAAGGATGGGTTTTTGAAGATGCAGACAATGGGAGTACCAGATTTTGTAATTTG
GGTATCCTCTGCGAAAGAAGACTGTGAAAGTGACTGTTTAAGTAACTGTTCCTGCATGGCATATTCATACTAC
ACAGGCATTGGTTGTATGCATTGGAATAGAAGCTTAATTGATATTCAAGAATACTACATGGATGGGGCGGCTG
ATTTGTTCATTCGTCTTGCCTACTCCGAACTTGGTAACATAGTTCTTTTCAACCTTAAGCTTTCAATTTTTTAGCT
TATTGTTTTGGACAACAAGGAGTTGAAATTTTGAAGTCTTCTAGGCTTTGAAGAATATAACCAAGAAAATCCT
AATATAATCTGCAATACCATTGAAGTAGTCAAATTCTCATATAGTGCTTTCACTTCCATGGAAAGAAAGATATG
CTCAAGAACTGTCTATTGCTTGTACATGTTTTGCAGCTGCAAATGACAAGAAAGACTTCCCTGTAGCAGCTATT
GCAATCACAGTTTCGATAGGCTCAATAATAGTTATCTTATGTGGATATCTTTTCTGGAAATTGTTGGCTAAGCAC
AGAGGTAATTAATCAAATGAGATTTTAGAGAACACAAACTAGAACTTCCATTTCTGCTAATTGTTTGATTTCTG
AATGTCCATTCTAAACCAGAAAGAAAGAGGAAAAAAGAAGCATTCTTAAGAGAAGCATCTTCAAAATTTTAC
CAAGGCGGCATGATTAAAGATGACATCAATCAAGTCAAAATTGAAGATATCACCTTGTACAGCTTTGACATGT
TAGCAAATGCAACTGACAGATTTCACTCAGCTAGCAAGCTGGGGCAAGGAGGCTTTGGTCCAGTCTACAAAG
TAATGTTTGTTGACACATCTCTGTAGTGTTCCTGACAAGTGCAAATAATACGAAATAAATTAGATTGATTATCTG
CAGGGAAAATTGCCAGATGGACAAGAAATTGCAGTAAAAAGGCTTTCACAGTCTTCTGGTCAGGGGGAACA
GGAGTTTATGAATGAGGTGGTCGTGATTTCTAGACTACAACATCATAATCTTGTTAGACTCCTCGGCTGCTGCA
CAGAAAGAGGGGAAAAGATGCTGGTGTATGATTTCATGCCCAATAGAAGCTTAGATGCATATCTTTTTGGTCA
GTGCAGAACTTTCTCTTATTGGTTTTGCCTGCCCTTCTAGTTAAGTACTTCTAGACTCAAACGAAAACTGTAAA
TCCATCATACATTTCTCGGTGATACCTTTCAGGTTCACACCAGGAAAGGTTCCTTGATTGGAGTAAACGAGCC
ATCATTATTGAAGGAACTGGTCGAGGCCTCCTTTACCTTCACAGAGACTCAAGACTACGAATTATTCATAGAG
ATCTGAAGGCAAGCAACATCCTGTTGGATGAATACCTAAACCCAAAAATTTCGGATTTTGGCATGGCGAGGAT

TTTTGGAGGAAATCAAAACCAGGCACGCACTATAAGAGTTGTTGGCACATAGTAAGTTGACATTGCTTCCTTT
CCATGGATGAACTGTCATCAATTGTCATTGAGTTTCTGTTTAATATGTTATGTAGTGGTTACATGGCCCCTGAAT
ATGCAATGCACGGAAGATTCTCAGAAAAATCAGATGTCTACAGCTTCGGTGTGTTGATCTTGGAAATTGTCAG
TGGAAGGAAGAACTCTAGTTTTTATGATGATGAAGATGAACTGACTCTACTTGCATATGTAAGTTCATTCTTGC
AACTATGAAGCTTTAATGTTCAGCTAGCTCATCAAACCAAAAAAATAGAGGAGAAAATTATGAAACTTCTGA
AAATAATATTTTCGCTGTTTCACAATGTCTTTGTTCTTTAGGCATGGAAGTTGTGGAACGAAAACAATATCATA
AAATTGATAGACCCCAAAATATTTGATTCAAGCTTCGAAAAACAGATGGTGAGATGTGTACATACTGGATTATT
ATGTGTTCAAGAATATGCAGAAGATAGGCCAAATGTCTCCACAGTTCTGTCAGTGCTCACGAGTGATATCGCT
GAACTACCTACTCCTAAACAACCTGCATTTACCGGAGGGCATGCTTCACCACAGCAAGGATCTTCTAAAAGC
CAAGGGTCCGTGAATGCTGATACCATAACTGTATTGGAACCACGA

>A157_02G016860.1_(*StRDA2C*)

ATGAGCTTATATCGATCATTTCTTGTTTTTCTCTGTTGCTTTTATGTAGTTTTTTCTGGTGCCAATGCTTCAGACAT
CATTACCAGTAGCGAACCCGTCAGGGACTCGGAAACTGTATTTTCCAGTGGTAAAAGATTTAAACTGGGATTT
TTCAGCCCAGGGAATTCTGCAAATCGTTATGTAGGGATTATGTTTAATCTACCATCACCAACACCAACTGCAGT
ATGGGTAGCTAACAGAGACAAGCCTATAAATGATTCTAGCGGAATATTCACAATATCAGATGATGGCAACCTT
GTAATCTTGAATGGACAGAAGGAGGTAATTTGGTCATCCAGTATTTCAAACTCCATGAAGAATTCCACTGCTC
AGCTCTTGGATACTGGCAACTTAGTCTTGAAAGACAGCTCAAATGGAAGAGTTATATGGGAAAGCTTTCAATA
TCCTACGGATTCTTTCTTACAGCTCATGAGAATGGGCATTTATAATAGTACTAACACAGCATCTCTCCTGAAAT
CATGGAGAAGTCCTGATGATCCATCTGTTGGGAGTTTCTCAGGTGGAATTCAACTTCAATACATCCCCCAGGC
TTTTATTTGGAATAACACCGCTCCTTACTTGCGTAGTAGTCCATGGAATAAACAGATCTATATTGGAATACCAG
AAATGATATCTTCCTACCGCTCTGGTTATGACCTCTTTGCTGATAATGTTGGCACCACATACCTAACTTATTCCC
ATGGAAATCAGTCTTTGATATTCTATTATTCCTTGAATTCAACAGGGTCTTATCAGGCGAAGGTTTGGGATCAA
AGTAAGAAGGATTGGGTGGTAACATGGGCAATTCCCCGCACTGAGTGTGATTTTTATGCTAAGTGCGGGACAT
TTGGAAGCTGTAATCCAAAGAATTCTCCAATATGCAGTTGTATACAAGGTTTTAAACCAAAACATGAAGGAG
AATGGGAGAAAGGAGATTGGTCTGGTGGATGCATCAGAACGACTGCACTACACTGTGAAAGGAACAGAAAT
GATGTTGAGAAGGGAAAGCAGGACGGGTTTTTGAAGCTGCAGACAATGGGAGTGCCAGATTTTGTAATTTCG
GCATCCTCAGCGAAAGAAGACTGTGAGAGTGACTGTTTGAATAACTGTTCCTGCACGGCATATTCATACTACT
CAGGCATAGGTTGTATGCATTGGAATAGAACTTTAATTGATATTCAACAATACTCCATGGATGGGGCGGCTGAT
TTGTTCATTCGCCTTGCCTACTCCGAACTTGGTAACATACTTCTTTTAACCTTAATCTTTCAGTTGTTTAGCTTAT
TTTCTTGGAACAACAAGAGTTGAAATTAGAAGTCTTATAATATTTGAAGAATATAACCAAGAAACTCATAGTA
AAAATCTACAATACCATCGAAGTAGTCTCAAATTCTCATATAGTGCTTTCACTTCCATGGAAAGAAAGATATGC
TCAAGAACTGTCTATGGCGCTCATACATGTTTTGCAGCTGCAAATGAAAAGAAAAACGTTCCTGTAGCAGCTA
TTGCAATCACAGTCTCAATAGGCTCAATAACAGTTATCTTATGTGGATATCTTTTCAGGAAATTGTTGGCTAAC
CACAAAGGTAATTAATCAAATGAGATTTTCGATAACACAAACTAGAACTTCTATGTCTAAAACTTGTTTGATTT
CTGAATGTCCACTCTAAACCAGAAAGGAAGAGGAAAAATGAAGCACTCTTAAGAGAAGCATCTCCAAAATT
TTACCAAGAAGGCATGATTAAAGATGACATCAACCAAGTCAAAATTGAAGACATCACCTTGTACAGCTTTGA
CATGTTAGCAAATGCCACTGACAGTTTTCAATCAGCTAGCAAGCTGGGGGAAGGAGGCTTTGGTCCAGTCTA
CAAAGTAATGTTTCTTGATGCATCTCTGCAGTGTTCTGAAAAATGTACACAATATAAAATAAATTTGATCAATT
ATCTGCAGGGGAAATTGCCAGATGGACAAGAAATTGCAGTAAAAAGGCTTTCACAGTTTTCTGGTCAGGGGC
TACAGGAGTTTATGAATGAGGTCGTCGTGATTTCTAGACTACAACATCGTAATCTTGTTAGACTCCTTGGCTGT
TGCACAGAGAGAGAGGGGAAAAGATGTTGGTTTATGATTTCATGCCAAATAGAAGCTTAGATACATATCTTTTTG
GTCAGTGCAGAACTTTCTTTCATTGGTTTTGCCTGTCCTTCAACTTAAGTACATTTAGACTCATAAGAAAACTG
TATTTCCTTAAGGAAACAAAATCCATCATACATTTCTTGGTGATAACTTTTCAGGTTCACACCAGGAAAAGTTC
CTTGATTGGAGTAAACGAGCTATCATTATTGAAGGAACTGGTCGAGGCCTCCTTTACCTTCACAGAGACTCAA

GACTACGAATTATTCATAGAGATCTGAAGGCAAGCAACATCCTGTTGGATGAATACCTAAACCCAAAAATTTC
GGATTTTGGCACGGCGAGGATCTTTGGAGGCAATCAAGATCAGGCCAACACTATAAGAGTTGTTGGAACATA
GTAAGTTTGTGCTGCTTCCTTCATTTTCTCATGGATGAACTGGCATCATTTGTCATTGAGTTTCTTCTTTAATATG
TTATGCAGTGGTTACATGGCCCCTGAATATGCAATGCATGGAAGATTCTCAGAAAAATCAGATGTCTACAGCT
TCGGTGTGTTGTTATTGGAAATTGTTAGTGGAAGGAAGAACTCTAGTTTTTCTGATGATGAAGATGAACTGACT
CTACTTGCATATGTAAGTTCCTTCTTGCAACTATGGAACTTAAATGGTTTTGCTTACTCATCAAACCAAAAAAA
AAAAAAAGGAGAAAAATTATGAAACTTCTGAAAATAATATTTTTGCTGTTTGACAATGTCTCTGTTCTTCAGG
CATGGAAGTTGTGGAATAAAAACAATATTGTAAAATTGATAGACCCCAAAATATTTGATTCAAGCTTCGAAAA
AGAGGTTGTGAGATGTGTACATATTGGATTATTATGTGTTCAAGAATATGCAGAAGATAGGCCAAATGTCTCCA
CAGTTCTATCAATGATCACTAGTGATAACGCTGAACTACCTACTCCTAAACAACCTGCTTTTACCAGAGGACA
TGCTTCACCACAGCCAGGATCTTCTAAAAGAGAAGGTTCGGTGAATGCTGATACCATAACTGTATTGGAACCA
CGA

>A157_02G016820.2_(*StRDA2D*)

ATGAAATTAAGCACAAGAGAGATACTTCTGTTTCTTTCTATATCAACTGCTTCAGGTATCATTAGCACCAATAA
ATTCCTGCGAGATTCAGAAACTTTAGTCTCCAATGACAAAAGATTCATATTCGGGTTCTTTAGTCCTGAAAATT
CGACGAATCGTTATGTTGGTGTTATGTTTAATGTCCAACCACCAACTGTTGTATGGGTTGCCAACAGAGAGAA
ACCTTTACAGGATTCTAGTGGAAGAGTGACAATATCTGATGATGGAAATCTTGTAATCTTGAATTCACAGAATA
GGAGTATATGGTCATCAAATATTTCACCAGCTGTGAGAAATTCCACAGCGCAGATCTTGGATACTGGAAACTT
AGTTTTGAACGATAGCTTCAATGGGAGGGTTCTCTGGGAAAGTTTTCGGGATCCTTCAGATTGCTTCTTGCAG
ACCATGAAAATTGGCGTTGATGTAAGTACTAACACGACAAATCTGCTGAAATCATGGATAAGTCCTTCAGATC
CATCTGTTGGGAGTTTCTCAGTTGGTATTCAACCTGAAACAGTTCCCCAGATTTCCATATGGAAGAATGGGAA
ACCTCATTGGCGTAGTGGTCCATGGAATAAACAGGTTTTCATTGGGGTACCAGACATGACTTCATTCTATCTCA
ATGGATTTGATCTAGTTAATGACAACAAGGGCACCGTGTACCTTACCTATTTATATGCAAATCAGATTGAGCTG
ACGTTTTTCACCTTGAACTCAACAGGGTTTTTGCAGCAGAAATATATGGATCCTAGTAAGAATGATTGGGAAG
TAACATGGGAATTTCCTGCAACTGAGTGTGATTTTTATGGAAAATGTGGACCTTTTGGAAGCTGTGATCCTACA
AGCTCACCAATCTGTTCTTGTTTAGAGGGATTTAGGCCGACAAGTGAAGAGGAATGGGGAAAAGGAAACTG
GACTCGTGGATGCAACAGAAAGTCCATGTTAGAGAGCGAAAGAAACAGTTCTAACCTTGAGCAAGGGAAGC
AAGATTGGTTTCTGAAGCTGCAGTCAATGAAAGTGCCGGATTCTGCTATTTGGGTACCTTTTGCAGATGAAGA
TTGTGTTAACGGTTGCTTGAGGAATACTTCATGCATAGCTTATTCATACTACACAGGCATAGGATGCATGCATT
GGGAAGGAAGCTTACTTGATGTTCAGAAATTCTCCATCGGTGGGGCAGATTTATTCCTCCGCCTTTCATACTCT
GAGCGTGGTAAGACCTGCATTTCAGAACTATAATATAAGCTATAAAACTCTGCTTGCCAATGACATATGTATAA
TGGACACTTATTTTGTAATGCAGATCAAAAGAGAGAATATAAAGTAGTCATTGCCATCATAGTCCCAGTAGGC
TCAATAATTCTTGCCATTTTCGGATACATTTCCTGCAAATATGTAGCTAAGCGCAGAGGTAACAAGCTTATCTG
AAACATTAGAAAATCCATAGTAGCTTTAATCATACGAATCACTTTATCTCTAATGTCCTCTTGAATCAGGATGG
AAGAGAATGAGTAAGATCTTATCAAGTGAATCATCGCCAAACTATTACAAGGAAGACAAGATTACAGAGGAC
ATCAATCAAGCTAAATTGGAAGAACTGCTTGTATACAACTTTGATATCTTAGCAAACGCAACTGAGAATTTTC
ATCTGTCCAGCAAGCTTGGACAGGGAGGTTTTGGTCCAGTTTACAAAGTAAGGATTTGTTTGATTGAGTGTCTT
ACAATTGTTTTTAAGAAAACATAACCAAGTATATGATATAATTGCAGGGGAAATTGCCAGATGGACAAGAGAT
TGCTGTGAAAAGGCTTTCACAGTCTTCTGGTCAGGGGCTGCAGGAGTTCATGAATGAGGTTGTGGTGATTTCA
AAACTTCAACATCGTAATCTTGTTAGACTTTTTGGGTGCTGCATAGAAAGAGGGGAAAAGATTCTGGTTTATG
AATACATGCCAAAAAGAAGCTTGGATGCCTATCTCTTTGGTTCGTTATGATTAGTTTTCTTTTATTTTCCATAGTC
TAACTAAAACAAACTTTTCTTTAATTTAAACTTGTCAAATTCGCGATGTATTTTCAGGGTCACAACAACAAGA
GGAAGAGTTCCTGGATTGGAGTAAACGTGTGATCATTATTGAGGGAATTGGTCGAGGCCTTCTTTACCTTCAC
AGGGATTCAAGACTAAGGATTATTCATAGGGATTTAAAGGCCAGCAACATTTTGTTGGATGAATACCTGAACC

CCAAAATTTCAGATTTTGGGATGGCAAGGATTTTTGCAGGCAACCAAGATCAGGCCAACACAAGCAGAGTAG
TTGGAACCTAGTAAGTTCTTGTATCTTTCCCTTTTATCTTTTCATGAATGAAAAGAACACAAAAAGTAAACATT
CAAAATTATAACATGATGCAGTGGTTACATGGCACCTGAATATGCAATGGAAGGAAGATTCTCAGAAAAATC
AGATGTTTATAGCTTTGGAGTATTGTTATTGGAAATTATAAGTGGAAGGAGGAACACTAGCTTTCACCAAGATG
ATGGTGCATTAAGCCTGCTAGCATTGGTAAGTTCTTATGAAAATTAAGCTCCAATTCATATGAAATGTCAACTT
TTCATAGTTCAAGTTTCCAATGTTTTGTCAACGTGCTTGCTCTTCAGGCGTGGAAATGTTGGATTGGAAACAAG
ATTGTGGAATTGGTTGATCCCAAGATAACTGACCTGCACCTTGAAAAAGAAATGGTGAGATGTGTACAAGTT
GGATTATTATGTGTACAAGAATATGCAGAAGACAGACCAAATGTCTCCACAATTTTGTCTATGCTCACCAGGG
AAATTGATGATTTACCAAGTCCTAAACAACCTGCATTTACAACAAGACCGAGCTTTTCCAAAAAATGCACTTC
TAAATCTCAAGGCTCCATTAACAATGTTACCGTTACTATTATGGAAGGACGA
